# Low‐lying placenta is associated with increased risk of hemorrhage and severe maternal morbidity: A nationwide analysis

**DOI:** 10.1002/pmf2.70468

**Published:** 2026-09-29

**Authors:** May Abiad, Giulia Bonanni, Nikan Zargarzadeh, Ali Javinani, Scott A. Shainker, Anna M. Modest, George R. Saade, Kjersti Aagaard, Alireza A. Shamshirsaz

**Affiliations:** ^1^ Fetal Care and Surgery Center, Division of Fetal Medicine and Surgery, Boston Children's Hospital Harvard Medical School Boston Massachusetts USA; ^2^ Department of Obstetrics and Gynecology, Beth Israel Deaconess Medical Center Harvard Medical School Boston Massachusetts USA; ^3^ Department of Obstetrics and Gynecology Eastern Virginia Medical School Norfolk Virginia USA; ^4^ HCA Healthcare and HCA Healthcare Research Institute Nashville Tennessee USA; ^5^ HCA Healthcare, Texas Maternal Fetal Medicine Houston Texas USA

**Keywords:** low‐lying placenta, maternal outcomes, obstetric hemorrhage, placenta previa, severe maternal morbidity

## Abstract

**Background:**

Placenta previa (PP) is a well‐recognized obstetric complication with established maternal risks, particularly hemorrhage, that contribute significantly to maternal morbidity. In contrast, low‐lying placenta (LLP) has historically been grouped with previa in both coding and clinical practice, limiting our ability to study its imparted associated outcomes as a distinct entity. However, in 2017, coding revisions allowed for a parsed diagnosis of LLP and PP and enabled the current study of differentiated associated outcomes.

**Objective(s):**

To describe and compare the independent rates of hemorrhage and severe maternal morbidity (SMM) associated with LLP and PP relative to normal placentation.

**Methods:**

We conducted a retrospective analysis using the Nationwide Readmissions Database (NRD) from 2017 through 2019, reflecting the initial 3 years of coding for LLP. Patients with multifetal gestation, ectopic/molar pregnancies, and other placental abnormalities were excluded. The primary outcome was hemorrhage, defined using international classification of diseases (ICD)‐10 codes for ante‐, intra‐, and postpartum hemorrhage (PPH). SMM was defined using the Centers for Disease Control and Prevention's index, which includes a range of life‐threatening complications such as disseminated intravascular coagulation, hysterectomy, and shock. Baseline characteristics and maternal outcomes were compared using chi‐square tests for categorical variables and Kruskal–Wallis tests for continuous variables. Multivariable regression analysis was used to evaluate the risk ratios (RRs) of adverse outcomes across these groups.

**Results:**

Among 5,625,432 singleton deliveries, 11,486 were diagnosed with LLP and 21,356 with PP. Compared to normal placentation, LLP was associated with a significantly higher rate of hemorrhage (21.1% vs. 4.0%, *p* < 0.001), SMM (4.5% vs. 1.8%, *p* < 0.001), maternal blood transfusion (3.3% vs. 1.1%, *p* < 0.001), hysterectomy (0.2% vs. < 0.1%, *p* < 0.001), and hysterectomy within 30 days (0.2% vs. 0.0%, *p* < 0.001). LLP was also associated with longer median hospital stay (3 vs. 2 days, *p* < 0.001) and higher total charges ($21,410 vs. $17,024; *p* < 0.001). In adjusted analyses using normal placentation as the referent group, LLP remained independently associated with hemorrhage (adjusted relative risk [aRR], 4.72; 95% CI, 4.56–4.89), SMM (aRR, 2.16; 95% confidence interval [CI], 1.99–2.35), and transfusion (aRR, 2.61; 95% CI, 2.36–2.88). PP was associated with even higher risks of hemorrhage (aRR, 8.93), SMM (aRR, 4.22), and transfusion (aRR, 5.69) compared to those with normal placentation (all *p* < 0.001).

**Conclusion:**

With the institution of distinct coding for LLP in 2017, we were able to distinguish between patients with LLP and PP. While PP remains associated with the highest risk of poor maternal outcomes, patients with an antenatal diagnosis of LLP also have an increased risk of hemorrhage and SMM relative to those with normal placentation. These findings should be considered during the prenatal counseling and management of patients with LLP.

## 1 INTRODUCTION

Low‐lying placenta (LLP) and placenta previa (PP) are conditions characterized by placental implantation near or overlying the internal cervical os, and are important causes of obstetric hemorrhage [1]. LLP, defined as the placental edge being within 2 cm of the internal os without covering it, is commonly identified on mid‐pregnancy ultrasound and often resolves as gestation progresses [2]. In contrast, PP, in which the placenta partially or completely covers the cervical os, occurs in approximately 4–5 per 1000 births globally [3, 4], is a well‐established high‐risk condition associated with significant maternal morbidity, including hemorrhage, transfusion, and hysterectomy [5, 6]. Although LLP has traditionally been considered a lower‐risk entity, emerging evidence suggests that it may still be associated with adverse maternal outcomes, suggesting that LLP should be viewed as part of a broader spectrum of abnormal placental implantation rather than a benign variant [7, 8, 9].

Prior studies have reported increased risks of postpartum hemorrhage (PPH), preterm birth, and cesarean delivery among patients with LLP, even in cases where placental location improves over the course of pregnancy [10, 11]. LLP has also been associated with abnormal placentation and bleeding‐related complications, particularly in the presence of prior cesarean delivery or placenta accreta spectrum (PAS) disorders [12, 13]. However, much of the existing literature is derived from single‐center cohorts or relatively small retrospective studies, which limits the ability to evaluate uncommon but clinically important maternal outcomes [2, 8]. In addition, earlier large database studies have often been unable to distinguish LLP from PP, resulting in these conditions being analyzed together despite differences in clinical severity and management [1].

The introduction of distinct international classification of diseases (ICD)‐10 coding for LLP beginning in 2017 provides an opportunity to evaluate LLP as a separate diagnostic entity at the population level. Using the Nationwide Readmissions Database (NRD), we sought to quantify the association between LLP and maternal morbidity in a large nationally representative cohort of US deliveries. Specifically, we aimed to compare rates of hemorrhage and severe maternal morbidity (SMM) across patients with LLP, PP, and normal placentation. By evaluating these groups within the same population and excluding patients with PAS disorders, this study aims to better characterize the maternal risk associated with LLP and inform prenatal counseling and delivery planning.

## 2 METHODS

### 2.1 Study design and data source

This was a retrospective cohort study using the NRD for the years 2017–2019. The NRD is maintained by the Healthcare Cost and Utilization Project (HCUP) and is one of the largest publicly available, all‐payer inpatient care databases in the United States, representing approximately 60% of US hospitalizations. It enables linkage of patients across admissions within a given calendar year and includes detailed discharge‐level data on patient demographics, diagnoses, procedures, hospital characteristics, and resource utilization. As the NRD is de‐identified and publicly available, this study was exempt from institutional review board approval. The study follows the Strengthening the Reporting of Observational Studies in Epidemiology (STROBE) guidelines.

### 2.2 Study population

We first identified delivery hospitalizations using ICD‐10‐clinical modification (CM) diagnosis and procedure codes for delivery. A complete list of all ICD‐10 codes used for exposure, outcome, and covariate classification is provided in Table S1. We excluded cases with multifetal gestation, ectopic or molar pregnancies, spontaneous or elective abortions, other placental abnormalities (ICD‐10‐CM codes O43101–O4393), including placental transfusion syndromes, placental infarction, placental dysfunction, other placental disorders, and unspecified placental disorders, as well as deliveries missing key outcome variables. PAS disorders were retained in the cohort and included as a covariate in adjusted analyses. Participants were followed for 30 days after delivery. Deliveries occurring in December were excluded due to lack of 30‐day follow‐up in the NRD. Additional exclusions included hospitalizations with a length of stay of 0 or >60 days and admissions with unknown or conflicting modes of delivery.

### 2.3 Exposure

The primary exposure was placental location identified using ICD‐10‐CM diagnostic codes recorded at the delivery hospitalization and categorized into three mutually exclusive groups: LLP, PP, and normal placentation. PP included diagnoses indicating complete or partial coverage of the cervical os. The introduction of specific ICD‐10 codes for LLP in 2017 allowed, for the first time, large‐scale stratification of outcomes between these two entities. However, as these codes are based on provider documentation, they may not uniformly represent sonographic confirmation. Normal placentation included deliveries without ICD‐10‐CM diagnoses of LLP or PP recorded during the delivery hospitalization. Deliveries with other placental abnormalities (ICD‐10‐CM codes O43101–O4393) were excluded from the cohort, while PAS disorders were retained and included as covariates in adjusted analyses. Because the NRD does not include prenatal imaging data, normal placentation cannot be confirmed using sonographic findings and may include cases in which abnormal placentation was not documented. The NRD does not contain information on the timing of diagnosis, serial imaging, or resolution of LLP; therefore, LLP represents diagnoses documented at delivery without the ability to distinguish persistent from resolved cases.

### 2.4 Outcomes

The primary outcome was maternal hemorrhage, identified using ICD‐10 codes for antepartum, intrapartum, or postpartum hemorrhage. Secondary outcomes included SMM, hysterectomy, blood transfusion, shock, disseminated intravascular coagulation (DIC), venous thromboembolism or pulmonary embolism (VTE/PE), cesarean delivery, readmission within 30 days, emergency readmission, hysterectomy within 30 days, and resource utilization measures such as length of stay and total hospital charges. Cesarean delivery was defined as any cesarean delivery documented during the delivery hospitalization, regardless of indication. The dataset does not allow reliable differentiation between planned and unplanned cesarean deliveries or between hemorrhage‐related and non‐hemorrhage‐related indications. SMM was defined using the Centers for Disease Control and Prevention (CDC) criteria, which encompass 21 life‐threatening conditions including but not limited to blood transfusion, hysterectomy, eclampsia, sepsis, shock, DIC, acute myocardial infarction, and mechanical ventilation [14]. SMM was examined both as a composite outcome including transfusion and separately excluding transfusion to account for variability in transfusion practices. All outcome definitions and corresponding ICD‐10 codes are provided in Tables S1 and S2.

### 2.5 Covariates

Patient‐level variables included maternal age, admission type (elective vs. non‐elective), and primary payer (Medicare, Medicaid, private insurance, self‐pay, or other). Information on parity is not available in the NRD. Gestational age (GA) was stratified into clinically meaningful categories reflecting established prematurity thresholds (<32 weeks, 32–33 weeks, 34–36 weeks, ≥37 weeks) and analyzed as a categorical variable to account for the nonlinear relationship between GA and maternal outcomes. However, GA may also lie on the causal pathway between placental abnormalities and adverse outcomes, and adjustment may attenuate observed associations. Pregnancy history and complications including chronic and gestational hypertension, preeclampsia, prior cesarean delivery, pregestational and gestational diabetes, fetal growth restriction (FGR), preterm labor, preterm premature rupture of membranes (PPROM), and placental abruption were included in the analysis. Comorbidity burden was assessed using the Elixhauser Comorbidity Index, a validated measure developed by the Agency for Healthcare Research and Quality (AHRQ) that identifies 31 distinct chronic medical conditions using administrative codes (Table S3) [15]. The number of comorbidities was categorized as 0, 1, 2, or 3 or more, consistent with prior obstetric risk stratification studies. Hospital‐level covariates included hospital bed size (small, medium, or large), teaching status and location (urban teaching, urban non‐teaching, or rural), and ownership type (government nonfederal, private not‐for‐profit, or private investor‐owned).

### 2.6 Statistical analysis

Descriptive statistics were used to summarize baseline demographic, clinical, and hospital characteristics. Categorical variables were reported as frequencies and percentages and compared using chi‐square tests. All continuous variables were assessed for distributional assumptions and were reported as medians with interquartile range (IQR) if found to be skewed. Continuous variables were compared using the Kruskal–Wallis test. Multivariable log‐binomial regression models were constructed to estimate adjusted relative risks (aRRs) and 95% confidence intervals (CIs) for the primary and secondary maternal outcomes, with normal placentation serving as the referent group. Variables included in the models were selected a priori based on clinical relevance and literature precedence evaluating risk factors for maternal morbidity, and included maternal age, admission type, insurance status, prior cesarean delivery, GA, comorbidity burden, and hospital characteristics. To evaluate the potential impact of delivery mode on the association between placental location and maternal outcomes, sensitivity analyses were performed stratified by mode of delivery. Discharge‐level weights were not applied, as the primary objective was to estimate associations between placental location and maternal outcomes rather than weighted incidence estimates. All statistical analyses were performed using Stata version 18.0 (StataCorp), and a two‐sided *p* value < 0.05 was considered statistically significant. Missingness for key covariates was minimal, and no imputation was performed.

## 3 RESULTS

### 3.1 Baseline sociodemographic and clinical characteristics

The final study population included 5,592,240 patients with normal placentation, 11,486 with LLP, and 21,356 with PP (Figure 1).

**FIGURE 1:**
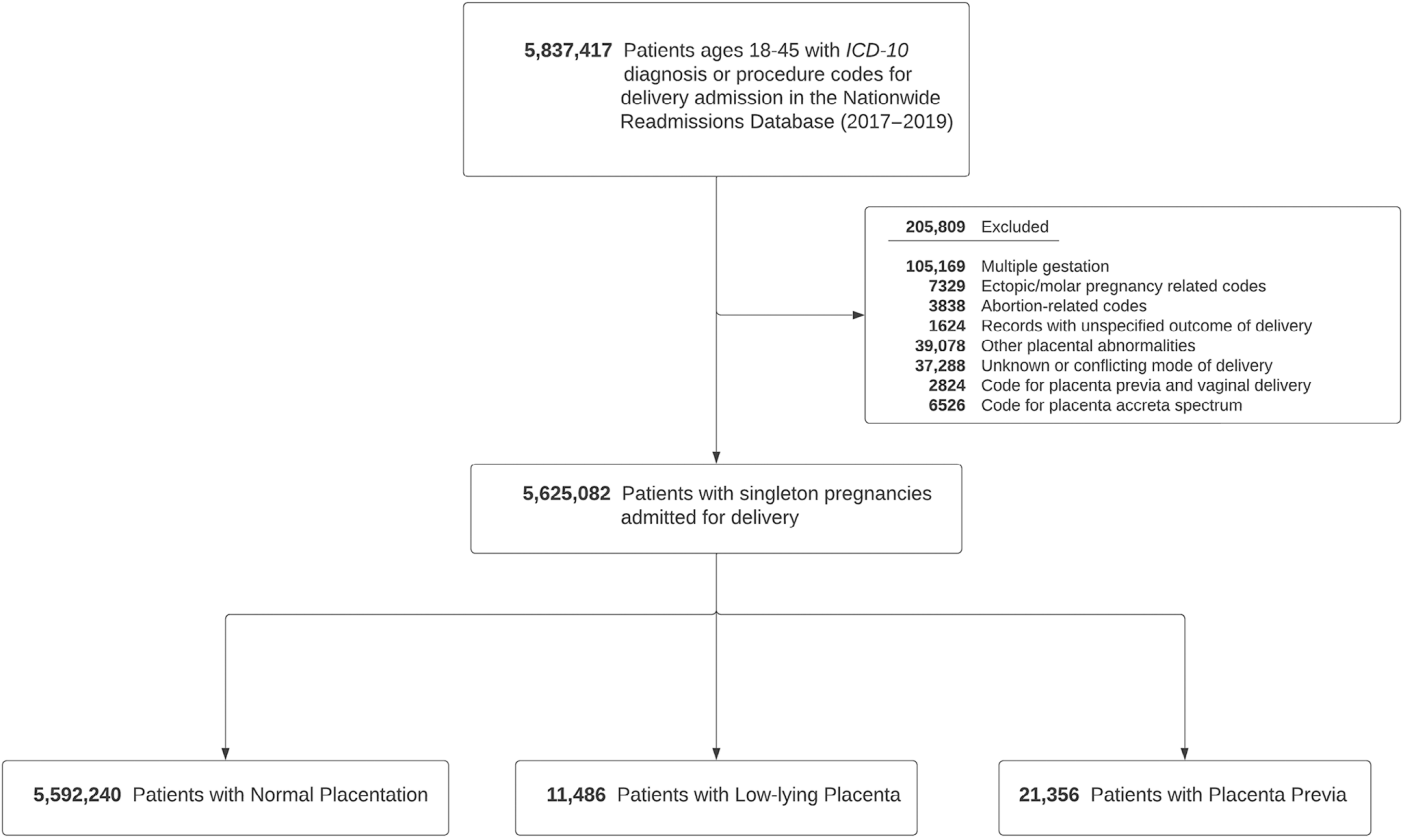
Patient selection flowchart.

Full sociodemographic and hospital characteristics are provided in Table 1. Compared with normal placentation, patients with LLP were older (median age 31 vs. 29; *p* < 0.001) and more likely to have a prior cesarean (22.2% vs. 17.8%; *p* < 0.001). Compared with LLP, patients with PP were older (median age 33 vs. 31; *p* < 0.001) and more likely to have a prior cesarean (26.6% vs. 22.2%; *p* < 0.001) (Table 1). LLP and PP cases were more frequently managed in large hospitals (57.2% and 60.3% vs. 55.2%; *p* < 0.001) and in urban teaching centers (76.0% and 78.7% vs. 71.3%; *p* < 0.001). Elective admissions were more common in PP (51.1%; *p* < 0.001) but similar between normal placentation (48.0%) and LLP (47.2%; *p* = 0.14).

**TABLE 1:** Baseline sociodemographic and clinical characteristics.

	Normal placentation	Low‐lying placenta		Placenta previa	
Variable	*N* = 5,592,240	*N* = 11,486	*p* value (normal vs. LLP)	*N* = 21,356	*p* value (LLP vs. PP)
Age, years, median (IQR)	29 (25–33)	31 (28–35)	<0.001	33 (29–36)	<0.001
Elective admission, *n* (%)	2,686,667 (48.0%)	5416 (47.2%)	0.14	10,921 (51.1%)	<0.001
Primary payer, *n* (%)			<0.001		0.005
Medicare	44,250 (0.8%)	90 (0.8%)		218 (1.0%)	
Medicaid	2,306,408 (41.2%)	3841 (33.4%)		7465 (35.0%)	
Private insurance	2,990,186 (53.5%)	7046 (61.3%)		12,755 (59.7%)	
Self‐pay	87,201 (1.6%)	165 (1.4%)		339 (1.6%)	
No charge	3062 (0.1%)	6 (0.1%)		20 (0.1%)	
Other	154,470 (2.8%)	326 (2.8%)		537 (2.5%)	
Bed size of hospital, *n* (%)			<0.001		<0.001
Small	954,616 (17.1%)	1848 (16.1%)		2843 (13.3%)	
Medium	1,551,998 (27.8%)	3063 (26.7%)		5637 (26.4%)	
Large	3,085,626 (55.2%)	6575 (57.2%)		12,876 (60.3%)	
Teaching status of urban hospitals, *n* (%)			<0.001		<0.001
Urban non‐teaching	1,150,567 (20.6%)	2127 (18.5%)		3655 (17.1%)	
Urban teaching	3,988,811 (71.3%)	8728 (76.0%)		16,807 (78.7%)	
Rural	452,862 (8.1%)	631 (5.5%)		894 (4.2%)	
Previous cesarean, *n* (%)	994,701 (17.8%)	2555 (22.2%)	<0.001	5683 (26.6%)	<0.001
Grand multiparity, *n* (%)	9417 (0.2%)	25 (0.2%)	0.20	63 (0.3%)	0.20
Obese, *n* (%)	594,215 (10.6%)	1225 (10.7%)	0.89	2029 (9.5%)	<0.001
Tobacco use, *n* (%)	291,230 (5.2%)	669 (5.8%)	0.003	1149 (5.4%)	0.093
Elixhauser comorbidities, *n* (%)			<0.001		0.001
None	3,059,201 (54.7%)	5078 (44.2%)		8991 (42.1%)	
1	1,330,573 (23.8%)	3133 (27.3%)		5902 (27.6%)	
2	764,448 (13.7%)	1982 (17.3%)		3938 (18.4%)	
3 or more	438,018 (7.8%)	1293 (11.3%)		2525 (11.8%)	
Gestational age at delivery, *n* (%)			<0.001		<0.001
Unspecified	9956 (0.2%)	16 (0.1%)		9 (0.0%)	
<32 weeks	88,364 (1.6%)	710 (6.2%)		2051 (9.6%)	
32–33 weeks	50,351 (0.9%)	313 (2.7%)		1467 (6.9%)	
34–36 weeks	338,966 (6.1%)	1212 (10.6%)		7783 (36.4%)	
≥37 weeks	5,038,900 (90.1%)	9127 (79.5%)		9785 (45.8%)	
Diabetes mellitus, *n* (%)			0.003		<0.001
None	5,085,777 (90.9%)	10,357 (90.2%)		18,899 (88.5%)	
Pregestational	59,860 (1.1%)	115 (1.0%)		278 (1.3%)	
Gestational	446,603 (8.0%)	1014 (8.8%)		2179 (10.2%)	
Hypertension, *n* (%)			<0.001		<0.001
None	5,110,567 (91.4%)	10,508 (91.5%)		20,068 (94.0%)	
Chronic hypertension	150,714 (2.7%)	371 (3.2%)		657 (3.1%)	
Gestational hypertension	330,959 (5.9%)	607 (5.3%)		631 (3.0%)	
Preeclampsia, *n* (%)	311,905 (5.6%)	733 (6.4%)	<0.001	830 (3.9%)	<0.001
HELLP syndrome, *n* (%)	14,220 (0.3%)	51 (0.4%)	<0.001	74 (0.3%)	0.17
Eclampsia, *n* (%)	14,220 (0.3%)	51 (0.4%)	<0.001	74 (0.3%)	0.17
Breech presentation, *n* (%)	180,267 (3.2%)	604 (5.3%)	<0.001	1863 (8.7%)	<0.001
FGR, *n* (%)	187,786 (3.4%)	633 (5.5%)	<0.001	992 (4.6%)	<0.001
Intrauterine infection, *n* (%)	134,909 (2.4%)	357 (3.1%)	<0.001	169 (0.8%)	<0.001
Preterm labor, *n* (%)	226,757 (4.1%)	931 (8.1%)	<0.001	3334 (15.6%)	<0.001
PPROM, *n* (%)	136,591 (2.4%)	657 (5.7%)	<0.001	860 (4.0%)	<0.001
Placental abruption, *n* (%)	59,337 (1.1%)	668 (5.8%)	<0.001	761 (3.6%)	<0.001
Uterine rupture, *n* (%)	3980 (0.1%)	7 (0.1%)	0.68	35 (0.2%)	0.013

Abbreviations: FGR, fetal growth restriction; HELLP, hemolysis, elevated liver enzymes, and low platelets; IQR, interquartile range; LLP, low‐lying placenta; PP, placenta previa; PPROM, preterm premature rupture of membranes.

### 3.2 Pregnancy outcomes

Adverse pregnancy outcomes were more frequent in LLP and PP (Table 1). Compared with normal placentation, preterm labor occurred in 8.1% of LLP versus 4.1% (*p* < 0.001), while rates were higher in PP compared with LLP (15.6% vs. 8.1%; *p* < 0.001). Preterm delivery before 37 weeks was common (20.5% in LLP vs. 9.9% in normal; *p* < 0.001), and remained substantially higher in PP compared with LLP (54.2% vs. 20.5%; *p* < 0.001). FGR was more frequent in LLP (5.5% vs. 3.4% in normal; *p* < 0.001), while rates in PP were slightly lower than LLP (4.6% vs. 5.5%; *p* < 0.001). Breech presentation affected 5.3% of LLP compared with 3.2% of normal (*p* < 0.001), and was more frequent in PP compared with LLP (8.7% vs. 5.3%; *p* < 0.001). Placental abruption occurred in 5.8% of LLP versus 1.1% of normal (*p* < 0.001), but was less frequent in PP compared with LLP (3.6% vs. 5.8%; *p* < 0.001). Intrauterine infection was highest in LLP (3.1% vs. 2.4% in normal; *p* < 0.001), and lower in PP compared with LLP (0.8% vs. 3.1%; *p* < 0.001). Uterine rupture remained rare (0.1% in LLP vs. 0.2% in PP).

### 3.3 Maternal outcomes

Marked differences emerged in maternal outcomes across the three groups (Table 2, Figure 2). Hemorrhage occurred in 4.0% of deliveries with normal placentation, increasing fivefold in LLP (21.1%; *p* < 0.001). Compared with LLP, rates were further increased in PP (42.8% vs. 21.1%; *p* < 0.001). SMM rose from 1.8% deliveries in normal placentation to 4.5% in LLP (*p* < 0.001), and was higher in PP compared with LLP (9.4% vs. 4.5%; *p* < 0.001). When transfusion was excluded, SMM remained elevated (0.84% in normal vs. 1.71% in LLP; *p* < 0.001), and higher in PP compared with LLP (2.96% vs. 1.71%; *p* < 0.001). Transfusion rates were 1.1% in normal placentation and 3.3% in LLP, and increased further in PP compared with LLP (7.6% vs. 3.3%; *p* < 0.001). Rare but serious complications also followed this gradient. DIC increased from 2.0 per 1000 (normal) to 5.2 in LLP (*p* < 0.001) and 8.2 in PP (compared with LLP; *p* = 0.002); shock increased from 0.5 per 1000 (normal) to 1.6 in LLP (*p* < 0.001) and 5.1 in PP (compared with LLP, *p* < 0.001). VTE/PE increased from 0.7 per 1000 (normal) to 2.3 in LLP (*p* < 0.001), with similar rates in PP compared with LLP (2.2 per 1000; *p* = 0.84). Hysterectomy within 30 days was also higher in LLP compared with normal placentation (0.2% vs. 0.0%; *p* < 0.001), and higher in PP compared with LLP (1.3% vs. 0.2%; *p* < 0.001). Median length of stay rose from 2 days (IQR 2–3) in normal placentation to 3 days (IQR 2–3; *p* < 0.001) in LLP, with similar values in PP compared with LLP (3 days; IQR 3–4; *p* < 0.001). Median hospital charges increased accordingly: $17,024 in normal placentation, $21,410 in LLP (*p* < 0.001), and further to $29,563 in PP compared with LLP (*p* < 0.001)

**TABLE 2:** Maternal outcomes in patients with and without LLP and placenta previa, without PAS.

	Normal placentation	Low‐lying placenta		Placenta Previa	
	*N* = 5,592,240	*N* = 11,486	*p* value (normal vs. LLP)	*N* = 21,356	*p* value (LLP vs. PP)
Hemorrhage, *n* (%)	226,046 (4.0%)	2422 (21.1%)	<0.001	9144 (42.8%)	<0.001
Cesarean delivery, *n* (%)	1,744,771 (31.2%)	6281 (54.7%)	<0.001	21,356 (100.0%)	<0.001
SMM any, *n* (%)	98,762 (1.8%)	521 (4.5%)	<0.001	2013 (9.4%)	<0.001
SMM excluding transfusion, *n* (%)	47,150 (0.8%)	196 (1.7%)	<0.001	631 (3.0%)	<0.001
Hysterectomy, *n* (%)	1834 (0.0%)	18 (0.2%)	<0.001	269 (1.3%)	<0.001
DIC, *n* (%)	11,282 (0.2%)	60 (0.5%)	<0.001	175 (0.8%)	0.002
Shock, *n* (%)	2985 (0.1%)	18 (0.2%)	<0.001	108 (0.5%)	<0.001
VTE or PE, *n* (%)	4050 (0.1%)	26 (0.2%)	<0.001	46 (0.2%)	0.84
Length of stay, days, median (IQR)	2 (2‐3)	3 (2‐3)	<0.001	3 (3‐4)	<0.001
Blood product transfusion, *n* (%)	59,478 (1.1%)	377 (3.3%)	<0.001	1621 (7.6%)	<0.001
Readmission within 30 days, *n* (%)	68,554 (1.2%)	159 (1.4%)	0.12	302 (1.4%)	0.83
Emergency readmission, *n* (%)	60,746 (1.1%)	133 (1.2%)	0.46	278 (1.3%)	0.26
Hysterectomy within 30 days, *n* (%)	2156 (0.0%)	19 (0.2%)	<0.001	272 (1.3%)	<0.001
Total charges, $, median (IQR)	17,024 (11,137–26,447)	21,409.5 (13592–33,887)	<0.001	29,563 (19,276–45,036)	<0.001

Abbreviations: DIC, disseminated intravascular coagulation; LLP, low‐lying placenta; IQR, interquartile range; PAS, placenta accreta spectrum; PE, pulmonary embolism; PP, placenta previa; SMM, severe maternal morbidity (CDC‐defined); VTE, venous thromboembolism.

**FIGURE 2:**
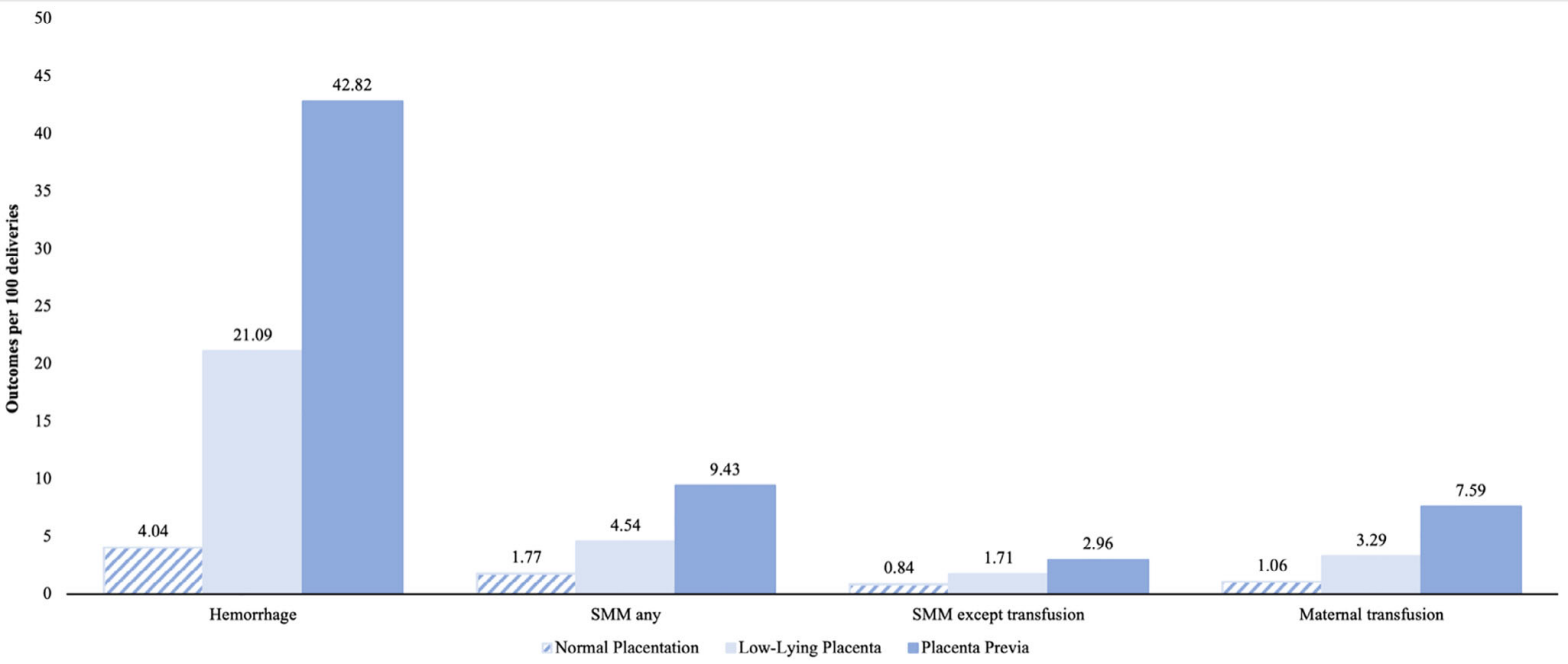
Absolute rates of maternal outcomes across comparison groups. SMM, severe maternal morbidity.

After adjustment for maternal, clinical, and hospital factors, both LLP and PP remained strongly associated with adverse maternal outcomes compared with normal placentation (Table 3). LLP was associated with more than a twofold increased risk of SMM (aRR, 2.16; 95% CI, 1.99–2.35; *p* < 0.001), while PP carried a fourfold increased risk (aRR, 4.22; 95% CI, 4.05–4.40; *p* < 0.001). LLP had nearly a fivefold risk of hemorrhage (aRR, 4.72; 95% CI, 4.56–4.89; *p* < 0.001), and PP had almost a ninefold risk (aRR, 8.93; 95% CI, 8.79–9.07; *p* < 0.001). LLP had over a twofold risk of transfusion (aRR, 2.61; 95% CI, 2.36–2.88; *p* < 0.001), while PP had nearly a sixfold risk (aRR, 5.69; 95% CI, 5.43–5.97; *p* < 0.001). LLP and PP were also associated with higher adjusted risks of DIC (aRR, 2.07 and 3.12; *p* < 0.001), shock (aRR, 2.37 and 7.34; *p* < 0.001), and VTE/PE (aRR, 2.72 and 2.51; *p* < 0.001).

**TABLE 3:** Risk ratios for maternal outcomes across comparison groups.

Outcome	Unadjusted RR (95% CI)		Adjusted RR (95% CI)^a^		*p* value**
	LLP	PP	LLP	PP	
Hemorrhage	5.22 (5.03–5.41)	10.59 (10.42–10.76)	4.72 (4.56–4.89)	8.93 (8.79–9.07)	<0.001
SMM any	2.57 (2.36–2.79)	5.34 (5.12–5.57)	2.16 (1.99–2.35)	4.22 (4.05–4.40)	<0.001
SMM excluding transfusion	2.02 (1.76–2.33)	3.50 (3.24–3.79)	1.69 (1.48–1.95)	2.83 (2.62–3.05)	<0.001
Maternal transfusion	3.09 (2.79–3.41)	7.14 (6.81–7.48)	2.61 (2.36–2.88)	5.69 (5.43–5.97)	<0.001
DIC	2.59 (2.01–3.33)	4.06 (3.50–4.71)	2.07 (1.61–2.67)	3.12 (2.69–3.62)	<0.001
Shock	2.94 (1.85–4.66)	9.47 (7.82–11.47)	2.37 (1.49–3.76)	7.34 (6.06–8.88)	<0.001
VTE or PE	3.13 (2.13–4.59)	2.97 (2.22–3.98)	2.72 (1.85–3.99)	2.51 (1.88–3.36)	<0.001

*Note*: Maternal transfusion refers to blood product transfusion.

Abbreviations: CI, confidence interval; DIC, disseminated intravascular coagulation; LLP, low‐lying placenta; PE, pulmonary embolism; PP, placenta previa; RR, relative risk; SMM, severe maternal morbidity (CDC‐defined); VTE, venous thromboembolism.

^a^Adjusted for maternal age, admission type, insurance status, prior cesarean delivery, gestational age, comorbidity burden, and hospital characteristics.

** *p* values correspond to comparisons of each exposure group (low‐lying placenta and placenta previa) with the reference group (normal placentation) within the regression model.

In sensitivity analyses stratified by mode of delivery, the association between LLP and adverse maternal outcomes persisted when compared to normal placentation (Table S4). Among cesarean deliveries, LLP was associated with higher risks of hemorrhage (aRR, 6.59; 95% CI, 6.30–6.89) and other adverse outcomes, with similar but smaller magnitudes observed among vaginal deliveries (hemorrhage aRR, 3.66; 95% CI, 3.46–3.88). Associations persisted across most outcomes, although estimates for shock and venous thromboembolism in vaginal deliveries did not reach statistical significance.

## 4 DISCUSSION

This study represents a retrospective analysis of the risks associated with a diagnosis of LLP and PP using a nationally representative database. Our findings support that LLP, while less severe than PP, is associated with significantly higher risks of composite SMM, or the individual outcomes of hemorrhage, blood transfusions, and hysterectomy compared to normal placentation. Notably, patients with a diagnosis of LLP had a fivefold increased risk of hemorrhage and over a twofold increased risk of SMM, underscoring the importance of vigilant monitoring and management. Intrauterine infection was modestly higher in the LLP group compared to normal placentation, but lower in PP. Delivery admissions for LLP were characterized by longer hospital stays and higher healthcare costs, reflecting the increased resource utilization associated with these cases. These findings establish LLP as a distinct and clinically significant condition warranting careful prenatal counseling and tailored management strategies.

Our findings are consistent with the existing studies indicating that both LLP and PP are associated with adverse maternal outcomes [16, 17]. Previous research has established PP as a high‐risk condition linked to significant maternal and neonatal morbidity, largely due to its strong association with PAS and preterm delivery [16, 18, 19]. In our study, PP was associated with a ninefold increased risk of hemorrhage and a fourfold increased risk of SMM compared with normal placentation. While LLP has frequently been associated with maternal risks similar to PP, its contribution to postpartum hemorrhage and PAS has remained less well quantified [20, 21]. A recent meta‐analysis by Bonanni et al. reported an increased risk of PPH among patients with LLP (RR, 2.10; 95% CI, 1.02–4.35; *p* = 0.05) [10]. While consistent with these findings, the stronger associations observed in our study may reflect differential ascertainment and coding within the NRD. Importantly, our study extends prior work by evaluating LLP separately from PP and demonstrating associations with SMM in a large nationally representative cohort.

Recent evidence has shown that individuals with high‐risk obstetric conditions who deliver at hospitals lacking the appropriate level of maternal care face a threefold increased risk of SMM [22]. Although our dataset does not include standardized classifications of maternal levels of care, the elevated risks associated with LLP and PP support enhanced prenatal surveillance including serial imaging to monitor changes in placental location and vascularity, as well as careful delivery planning. In addition, patients with LLP and those at risk for PAS should be referred to and deliver at hospitals equipped to provide subspecialty (Level III) or regional (Level IV) maternal care [23]. These centers offer access to multidisciplinary expertise and resources essential for managing obstetric emergencies and other high‐risk maternal complications.

Despite the insights provided by this study, several questions remain unanswered. Future research should investigate the mechanisms underlying the increased maternal morbidity associated with LLP including the potential roles of residual vascular changes and placental attachment abnormalities. Prospective studies evaluating strategies to reduce adverse maternal outcomes, as well as longitudinal assessments of neonatal outcomes and long‐term maternal outcomes, are needed.

The strengths of this study include its large, nationally representative sample and the use of robust statistical methods to adjust for potential confounders. The ability to distinguish between LLP and PP using distinct coding represents a significant methodological advancement, allowing for more precise risk stratification. However, several limitations should be acknowledged. First, the retrospective design and reliance on administrative data preclude causal inference and may introduce misclassification and ascertainment bias. Pregnancies complicated by LLP may undergo increased clinical surveillance, increasing the likelihood that complications are captured and LLP is documented, whereas incomplete coding of placental location at delivery may result in misclassification of some LLP cases as normal placentation, which would bias estimates toward the null. Consequently, the observed associations may either overestimate or underestimate the true magnitude of risk. Notably, the observed frequency of LLP in this cohort was lower than that of PP, suggesting potential under‐ascertainment of LLP in routine coding practices. Additionally, ICD‐10 coding is based on provider documentation rather than standardized sonographic criteria, and variation in diagnostic thresholds across institutions may further contribute to heterogeneity within the LLP group. As a result, the LLP category in this study likely represents a heterogeneous group with varying degrees of proximity to the internal cervical os. Additionally, the lack of granular clinical data, such as detailed imaging findings and operative notes, limits the ability to explore underlying mechanisms and individual variations in management. Information on parity is not available in the NRD, preventing stratification of outcomes by nulliparous versus multiparous patients. The severity of LLP (i.e., the exact distance from the cervical os) cannot be assessed, nor can we determine whether diagnoses were made antenatally or intrapartum, including for time‐sensitive complications such as placental abruption. Similarly, certain clinical conditions such as FGR may be undercoded in administrative datasets, as identification relies on provider documentation. Finally, because coding occurs at the time of hospitalization, complications may increase the likelihood that LLP is documented, which could contribute to differential ascertainment. The findings may not be generalizable to populations outside the United States or to healthcare settings with different obstetric coding practices or levels of care.

## 5 CONCLUSIONS

This study highlights the substantial maternal health burden associated with a diagnosis of LLP and/or PP, emphasizing the need for heightened clinical awareness and delivery planning. Although PP remains more strongly linked to adverse outcomes, LLP is also associated with clinically meaningful risks that warrant serious clinical attention, enhanced prenatal surveillance, individualized counseling, and multidisciplinary planning tailored to patient risk. Delivery in settings with appropriate resources and expertise may help mitigate complications. Further research is needed to better define optimal management strategies and inform evidence‐based guidelines for patients with LLP.

## CONFLICT OF INTEREST STATEMENT

The authors declare no conflicts of interest.

## FUNDING INFORMATION

The authors received no specific funding for this work.

## Supporting information



Supporting Information
