# Supplementary material for: Low‐lying placenta is associated with increased risk of hemorrhage and severe maternal morbidity: A nationwide analysis
Source: Pregnancy (Hoboken). 2026 Sep 29;2(6):e70468. doi: 10.1002/pmf2.70468 (PMC13620832; doi:10.1002/pmf2.70468)
Supplement: Supplementary file 1 [file PMF2-2-e70468-s001.docx]

**Supplementary Table 1.** ICD-10 Codes for Inclusion, Exclusion, Covariates, and Outcome Variables

| Variable | ICD-10 Codes |
| --- | --- |
| **Inclusion Criteria** | |
| Delivery Hospitalization | Diagnosis Codes: Z37 Z370 Z3800 Z3801 Z381 Z382 Z371 O80 O82 O7582 Z372 Z373 Z374 Z3750 Z3751 Z3752 Z3753 Z3754 Z3759 Z3760 Z3761 Z3762 Z3763 Z3764 Z3769 Z377 Z3830 Z3831 Z384 Z385 Z3861 Z3862 Z3863 Z3864 Z3865 Z3866 Z3868 Z3869 Z387 Z388  Procedure Codes: 10D00Z0 10D00Z1 10D00Z2 10D07Z3 10D07Z4 10D07Z5 10D07Z6 10D07Z7 10D07Z8 10E0XZZ |
| Low-lying Placenta | O4440 O4441 O4442 O4443 O4450 O4451 O4452 O4453 |
| Placenta Previa | O4400 O4401 O4402 O4403 O4420 O4421 O4422 O4423 O4410 O4411 O4412 O4413 O4430 O4431 O4432 O4433 |
| Cesarean Delivery | Diagnosis Codes: O82 Z3801 O7582 Procedure Codes: 10D00Z0 10D00Z1 10D00Z2 |
| **Exclusion Criteria** | |
| Ectopic/Molar Pregnancy | Diagnosis Codes: O000 O0000 O0001 O001 O0010 O00101 O00102 O00109 O0011 O00111 O00112 O00119 O002 O0020 O00201 O00202 O00209 O0021 O00211 O00212 O00219 O008 O0080 O0081 O009 O0090 O0091 O010 O011 O019 O020 O021 O0281 O0289 O029 O080 O081 O082 O083 O084 O085 O086 O087 O0881 O0882 O0883 O0889 O089  Procedure Codes: 10D20ZZ 10D24ZZ 10D27ZZ 10D28ZZ 10J20ZZ 10J23ZZ 10J24ZZ 10J27ZZ 10J28ZZ 10J2XZZ 10S20ZZ 10S23ZZ 10S24ZZ 10S27ZZ 10S28ZZ 10T20ZZ 10T23ZZ 10T24ZZ 10T27ZZ 10T28ZZ |
| Multiple Gestation | O30001 O30002 O30003 O30009 O30011 O30012 O30013 O30019 O30021 O30022 O30023 O30029 O30031 O30032 O30033 O30039 O30041 O30042 O30043 O30049 O30091 O30092 O30093 O30099 O30101 O30102 O30103 O30109 O30111 O30112 O30113 O30119 O30121 O30122 O30123 O30129 O30131 O30132 O30133 O30139 O30191 O30192 O30193 O30199 O30201 O30202 O30203 O30209 O30211 O30212 O30213 O30219 O30221 O30222 O30223 O30229 O30231 O30232 O30233 O30239 O30291 O30292 O30293 O30299 O30801 O30802 O30803 O30809 O30811 O30812 O30813 O30819 O30821 O30822 O30823 O30829 O30831 O30832 O30833 O30839 O30891 O30892 O30893 O30899 O3090 O3091 O3092 O3093 O3100X0 O3100X1 O3100X2 O3100X3 O3100X4 O3100X5 O3100X9 O3101X0 O3101X1 O3101X2 O3101X3 O3101X4 O3101X5 O3101X9 O3102X0 O3102X1 O3102X2 O3102X3 O3102X4 O3102X5 O3102X9 O3103X0 O3103X1 O3103X2 O3103X3 O3103X4 O3103X5 O3103X9 O3110X0 O3110X1 O3110X2 O3110X3 O3110X4 O3110X5 O3110X9 O3111X0 O3111X1 O3111X2 O3111X3 O3111X4 O3111X5 O3111X9 O3112X0 O3112X1 O3112X2 O3112X3 O3112X4 O3112X5 O3112X9 O3113X0 O3113X1 O3113X2 O3113X3 O3113X4 O3113X5 O3113X9 O3120X0 O3120X1 O3120X2 O3120X3 O3120X4 O3120X5 O3120X9 O3121X0 O3121X1 O3121X2 O3121X3 O3121X4 O3121X5 O3121X9 O3122X0 O3122X1 O3122X2 O3122X3 O3122X4 O3122X5 O3122X9 O3123X0 O3123X1 O3123X2 O3123X3 O3123X4 O3123X5 O3123X9 O3130X0 O3130X1 O3130X2 O3130X3 O3130X4 O3130X5 O3130X9 O3131X0 O3131X1 O3131X2 O3131X3 O3131X4 O3131X5 O3131X9 O3132X0 O3132X1 O3132X2 O3132X3 O3132X4 O3132X5 O3132X9 O3133X0 O3133X1 O3133X2 O3133X3 O3133X4 O3133X5 O3133X9 O318X10 O318X11 O318X12 O318X13 O318X14 O318X15 O318X19 O318X20 O318X21 O318X22 O318X23 O318X24 O318X25 O318X29 O318X30 O318X31 O318X32 O318X33 O318X34 O318X35 O318X39 O318X90 O318X91 O318X92 O318X93 O318X94 O318X95 O318X99 Z372 Z373 Z374 Z3750 Z3751 Z3752 Z3753 Z3754 Z3759 Z3760 Z3761 Z3762 Z3763 Z3764 Z3769 Z377 Z372 Z373 Z374 Z3750 Z3751 Z3752 Z3753 Z3754 Z3759 Z3760 Z3761 Z3762 Z3763 Z3764 Z3769 Z377 Z3830 Z3831 Z384 Z385 Z3861 Z3862 Z3863 Z3864 Z3865 Z3866 Z3868 Z3869 Z387 Z388 |
| Abortion (spontaneous/induced) | Diagnosis Codes: O030 O031 O032 O0330 O0331 O0332 O0333 O0334 O0335 O0336 O0337 O0338 O0339 O034 O035 O036 O037 O0380 O0381 O0382 O0383 O0384 O0385 O0386 O0387 O0388 O0389 O039 O045 O046 O047 O0480 O0481 O0482 O0483 O0484 O0485 O0486 O0487 O0488 O0489 O070 O071 O072 O0730 O0731 O0732 O0733 O0734 O0735 O0736 O0737 O0738 O0739 O074  Procedure Codes: 10A00ZZ 10A03ZZ 10A04ZZ 10A07Z6 10A07ZW 10A07ZX 10A07ZZ 10A08ZZ |
| Unspecified Outcome of Delivery | Z379 |
| Cord anomalies | O694XX0 O694XX1 O694XX2 O694XX3 O694XX4 O694XX5 O694XX9 O43121 O43122 O43123 O43129 |
| Other placental abnormalities | O43101 O43102 O43103 O43109 O43111 O43112 O43113 O43119 O43811 O43812 O43813 O43819 O43891 O43892 O43893 O43899 O4390 O4391 O4392 O4393 |

| Variable | ICD-10 Codes |
| --- | --- |
| **Covariates** | |
| Placenta Accreta Spectrum (PAS) Disorders | O43211 O43212 O43213 O43219 O43221 O43222 O43223 O43229 O43231 O43232 O43233 O43239 |
| Fetal Growth Restriction | O365 Z364 O365910 O365911 O365912 O365913 O365914 O365915 O365919 O365920 O365921 O365922 O365923 O365924 O365925 O365929 O365930 O365931 O365932 O365933 O365934 O365935 O365939 O365990 O365991 O365992 O365993 O365994 O365995 O365999 Z364 |
| Diabetes | Pre-gestational Diabetes Mellitus: O24011 O24012 O24013 O24019 O2402 O2403 O24111 O24112 O24113 O24119 O2412 O2413 O24311 O24312 O24313 O24319 O2432 O2433 O24811 O24812 O24813 O24819 O2482 O2483 O2483  Gestational Diabetes Mellitus: O24410 O24414 O24415 O24419 O24420 O24420 O24424 O24425 O24425 O24429 O24429 O24430 O24434 O24435 O24439 |
| Hypertensive Disorders of Pregnancy | Chronic Hypertension: O10011 O10012 O10013 O10019 O1002 O1003 O10411 O10412 O10413 O10419 O1042 O10911 O10912 O10913 O10919 O1092 O1093  Gestational Hypertension: O131 O132 O133 O134  Pre-eclampsia: O111 O112 O113 O114 O119 O1400 O1402 O1403 O1404 O1404 O1410 O1412 O1413 O1414 O1490 O1492 O1493 O1494  Eclampsia: O1500 O1502 O1503 O151 O152 O159 |
| Prior Cesarean Delivery | O3421 O34211 O34212 O34218 O34219 O3422 O6641 |
| Preterm Labor | O6000 O6002 O6003 O6010X0 O6010X1 O6010X2 O6010X3 O6010X4 O6010X5 O6010X9 O6012X0 O6012X1 O6012X2 O6012X3 O6012X4 O6012X5 O6012X9 O6013X0 O6013X1 O6013X2 O6013X3 O6013X4 O6013X5 O6013X9 O6014X0 O6014X1 O6014X2 O6014X3 O6014X4 O6014X5 O6014X9 O6020X0 O6020X0 O6020X1 O6020X1 O6020X2 O6020X2 O6020X3 O6020X3 O6020X4 O6020X4 O6020X5 O6020X5 O6020X9 O6020X9 O6022X0 O6022X0 O6022X1 O6022X1 O6022X2 O6022X2 O6022X3 O6022X3 O6022X4 O6022X4 O6022X5 O6022X5 O6022X9 O6022X9 O6023X0 O6023X0 O6023X1 O6023X1 O6023X2 O6023X2 O6023X3 O6023X3 O6023X4 O6023X4 O6023X5 O6023X5 O6023X9 O6023X9 |
| Placental Abruption | O45001 O45001 O45002 O45002 O45003 O45003 O45009 O45009 O45011 O45011 O45012 O45012 O45013 O45013 O45019 O45019 O45021 O45021 O45022 O45022 O45023 O45023 O45029 O45029 O45091 O45091 O45092 O45092 O45093 O45093 O45099 O45099 O458X1 O458X1 O458X2 O458X2 O458X3 O458X3 O458X9 O458X9 O4590 O4590 O4591 O4591 O4592 O4592 O4593 O4593 |
| Breech Presentation | O321 O641 O321XX0 O321XX1 O321XX2 O321XX3 O321XX4 O321XX5 O321XX9 O641XX0 O641XX1 O641XX2 O641XX3 O641XX4 O641XX5 O641XX9 |
| PPROM | O42011 O42012 O42013 O42019 O42111 O42112 O42113 O42119 O42911 O42912 O42913 O42919 |
| Gestational Age | Z3A00 Z3A01 Z3A08 Z3A09 Z3A10 Z3A11 Z3A12 Z3A13 Z3A14 Z3A15 Z3A16 Z3A17 Z3A18 Z3A19 Z3A20 Z3A21 Z3A22 Z3A23 Z3A24 Z3A25 Z3A26 Z3A27 Z3A28 Z3A29 Z3A30 Z3A31 Z3A32 Z3A33 Z3A34 Z3A35 Z3A36 Z3A37 Z3A38 Z3A39 Z3A40 Z3A41 Z3A42 Z3A49 |
| Hemorrhage | O46001 O46002 O46003 O46009 O46011 O46012 O46013 O46019 O46021 O46022 O46023 O46029 O46091 O46092 O46093 O46099 O468X1 O468X2 O468X3 O468X9 O4690 O4691 O4692 O4693 O4450 O4451 O4452 O4453 O4410 O4411 O4412 O4413 O4430 O4431 O4432 O4433 O670 O678 O679 O720 O721 O722 |
| Uterine Rupture | O7100 O7102 O7103 O711 |
| Disseminated Intravascular Coagulation (DIC) | D65 D688 D689 O45002 O45003 O45009 O45012 O45013 O45019 O45022 O45023 O45029 O45092 O45093 O45099 O46002 O46003 O46009 O46012 O46013 O46019 O46022 O46023 O46029 O46092 O46093 O46099 O670 O723 |

**Supplementary Table 2. CDC Severe Maternal Morbidity**

| **Type** | **Variable** | **Codes** |
| --- | --- | --- |
| **Diagnosis** | Acute MI | I2101 I2102 I2109 I2111 I2119 I2121 I2129 I213 I214 I219 I21A1 I21A9 I21B I220 I221 I222 I228 I229 |
|  | Aneurysm | I7100 I7101 I71010 I71011 I71012 I71019 I7102 I7103 I711 I7110 I7111 I7112 I7113 I712 I7120 I7121 I7122 I7123 I713 I7130 I7131 I7132 I7133 I714 I7140 I7141 I7142 I7143 I715 I7150 I7151 I7152 I716 I7160 I7161 I7162 I718 I719 I790 I791 |
|  | Acute Renal Failure | N170 N171 N172 N178 N179 O904 |
|  | ARDS | J80 J951 J952 J953 J95821 J95822 J9600 J9601 J9602 J9620 J9621 J9622 J9690 J9691 J9692 R0603 R092 |
|  | Amniotic Fluid Embolism | O88112 O88113 O88119 O8812 O8813 |
|  | Cardiac Arrest / Ventricular Fibrillation | I462 I468 I469 I4901 I4902 |
|  | DIC | D65 D688 D689 O45002 O45003 O45009 O45012 O45013 O45019 O45022 O45023 O45029 O45092 O45093 O45099 O46002 O46003 O46009 O46012 O46013 O46019 O46022 O46023 O46029 O46092 O46093 O46099 O670 O723 |
|  | Eclampsia | O1500 O1502 O1503 O151 O152 O159 |
|  | Heart Failure/Arrest during Surgery of Procedure | I97120 I97121 I97130 I97131 I97710 I97711 |
|  | Puerperal Cerebrovascular Disorders | A812 G450 G451 G452 G453 G454 G458 G459 G460 G461 G462 G463 G464 G465 G466 G467 G468 G9349 H340 H3400 H3401 H3402 H3403 I6000 I6001 I6002 I6010 I6011 I6012 I602 I6020 I6021 I6022 I6030 I6031 I6032 I604 I6050 I6051 I6052 I606 I607 I608 I609 I610 I611 I612 I613 I614 I615 I616 I618 I619 I6200 I6201 I6202 I6203 I621 I629 I6300 I63011 I63012 I63013 I63019 I6310 I63111 I63112 I63113 I63119 I6312 I63131 I63132 I63133 I63139 I6319 I6320 I63211 I63212 I63213 I63219 I6322 I63231 I63232 I63233 I63239 I6329 I6330 I63311 I63312 I63313 I63319 I63321 I63322 I63323 I63329 I63331 I63332 I63333 I63339 I63341 I63342 I63343 I63349 I6339 I6340 I63411 I63412 I63413 I63419 I63421 I63422 I63423 I63429 I63431 I63432 I63433 I63439 I63441 I63442 I63443 I63449 I6349 I6350 I63511 I63512 I63513 I63519 I63521 I63522 I63523 I63529 I63531 I63532 I63533 I63539 I63541 I63542 I63543 I63549 I6359 I636 I638 I6381 I6389 I639 I6501 I6502 I6503 I6509 I651 I6521 I6522 I6523 I6529 I658 I659 I6601 I6602 I6603 I6609 I6611 I6612 I6613 I6619 I6621 I6622 I6623 I6629 I663 I668 I669 I670 I671 I672 I673 I673 I674 I674 I675 I676 I677 I6781 I6782 I6783 I67841 I67848 I67850 I67858 I6789 I679 I680 I682 I688 O2250 O2252 O2253 I97810 I97811 I97820 I97821 O873 |
|  | Pulmonary Edema / Acute Heart Failure | I501 I5020 I5021 I5023 I5030 I5031 I5033 I5040 I5041 I5043 I50810 I50811 I50813 I50814 I5082 I5083 I5084 I5089 I509 J810 |
|  | Severe Anesthesia Complications | O29112 O29113 O29119 O29122 O29123 O29129 O29192 O29193 O29199 O29212 O29213 O29219 O29292 O29293 O29299 O740 O741 O742 O743 O8901 O8909 O891 O892 T882XXA T883XXA |
|  | Sepsis | A327 A400 A401 A403 A408 A409 A4101 A4102 A411 A412 A413 A414 A4150 A4151 A4152 A4153 A4154 A4159 A4181 A4189 A419 I76 O85 O8604 R6520 R6521 T8112XA T8144XA |
|  | Shock | O751 R570 R571 R578 R579 T782XXA T8110XA T8111XA T8119XA T886XXA |
|  | Sickle Cell Disease with Crisis | D5700 D5701 D5702 D57211 D57212 D57219 D57411 D57412 D57419 D57811 D57812 D57819 |
|  | Air and Thrombotic Embolism | I2601 I2602 I2609 I2690 I2692 I2693 I2694 I2699 O88012  O88013 O88019 O88.03 O88212 O88213 O88219 O8822 O8823 O88312 O88313 O88319 O8832 O8833 O88812 O88813 O88819 O8882 O8883 T80.0XXA |
| **Procedure** | Conversion of Cardiac Rhythm | 5A12012 5A2204Z |
|  | Blood Products Transfusion | 30233H1 30233L1 30233K1 30233M1 30233N1 30233P1 30233R1 30233T1 30233H0 30233L0 30233K0 30233M0 30233N0 30233P0 30233R0 30233T0 30230H1 30230L1 30230K1 30230M1 30230N1 30230P1 30230R1 30230T1 30230H0 30230L0 30230K0 30230M0 30230N0 30230P0 30230R0 30230T0 30240H1 30240L1 30240K1 30240M1 30240N1 30240P1 30240R1 30240T1 30240H0 30240L0 30240K0 30240M0 30240N0 30240P0 30240R0 30240T0 30243H1 30243L1 30243K1 30243M1 30243N1 30243P1 30243R1 30243T1 30243H0 30243L0 30243K0 30243M0 30243N0 30243P0 30243R0 30243T0 30250H1 30250L1 30250K1 30250M1 30250N1 30250P1 30250R1 30250T1 30250H0 30250L0 30250K0 30250M0 30250N0 30250P0 30250R0 30250T0 30253H1 30253L1 30253K1 30253M1 30253N1 30253P1 30253R1 30253T1 30253H0 30253L0 30253K0 30253M0 30253N0 30253P0 30253R0 30253T0 30260H1 30260L1 30260K1 30260M1 30260N1 30260P1 30260R1 30260T1 30260H0 30260L0 30260K0 30260M0 30260N0 30260P0 30260R0 30260T0 30263H1 30263L1 30263K1 30263M1 30263N1 30263P1 30263R1 30263T1 30263H0 30263L0 30263K0 30263M0 30263N0 30263P0 30263R0 30263T0 |
|  | Hysterectomy | 0UT90ZZ 0UT94ZZ 0UT97ZZ 0UT98ZZ 0UT9FZZ |
|  | Temporary Tracheostomy | 0B110Z4 0B110F4 0B113Z4 0B113F4 0B114Z4 0B114F4 |
|  | Mechanical Ventilation | 5A1935Z 5A1945Z 5A1955Z |

**Supplementary Table 3.** ICD-10 Codes for Elixhauser Comorbidities

| Variable | ICD-10 Codes |
| --- | --- |
| **Elixhauser Comorbidities** | |
| AIDS | B20 O98711 O98712 O98713 O98719 O9872 O9873 Z21 |
| Alcohol Abuse | F1010 F1011 F10120 F10121 F10129 F10130 F10131 F1013 F10139 F1014 F10150 F10151 F10159 F10180 F10181 F10182 F10188 F1019 F1020 F1021 F10220 F10221 F10229 F10230 F10231 F10232 F10239 F1024 F10250 F10251 F10259 F1026 F1027 F10280 F10281 F10282 F10288 F1029 F1094 F10950 F10951 F10959 F1096 F1097 F10980 G621 I426 K2920 K2921 K7010 K7011 O99310 O99311 O99312 O99313 O99314 O99315 |
| Deficiency Anemias | D501 D508 D509 D510 D511 D512 D513 D518 D519 D520 D521 D528 D529 D530 D531 D532 D538 D539 D630 D631 D638 D649 O99011 O99012 O99013 O99019 |
| Autoimmune disorders | A1801 A1802 A3984 A5441 A5442 L4050 L4051 L4054 L4059 L900 L940 L941 L943 M0500 M05011 M05012 M05019 M05021 M05022 M05029 M05031 M05032 M05039 M05041 M05042 M05049 M05051 M05052 M05059 M05061 M05062 M05069 M05071 M05072 M05079 M0509 M0510 M05111 M05112 M05119 M05121 M05122 M05129 M05131 M05132 M05139 M05141 M05142 M05149 M05151 M05152 M05159 M05161 M05162 M05169 M05171 M05172 M05179 M0519 M0520 M05211 M05212 M05219 M05221 M05222 M05229 M05231 M05232 M05239 M05241 M05242 M05249 M05251 M05252 M05259 M05261 M05262 M05269 M05271 M05272 M05279 M0529 M0530 M05311 M05312 M05319 M05321 M05322 M05329 M05331 M05332 M05339 M05341 M05342 M05349 M05351 M05352 M05359 M05361 M05362 M05369 M05371 M05372 M05379 M0539 M0540 M05411 M05412 M05419 M05421 M05422 M05429 M05431 M054320 M05439 M05441 M05442 M05449 M05451 M05452 M05459 M05461 M05462 M05469 M05471 M05472 M05479 M0549 M0550 M05511 M05512 M05519 M05521 M05522 M05529 M05531 M05532 M05539 M05541 M05542 M05549 M05551 M05552 M05559 M05561 M05562 M05569 M05571 M05572 M05579 M0559 M0560 M05611 M05612 M05619 M05621 M05622 M05629 M05631 M05632 M05639 M05641 M05642 M05649 M05651 M05652 M05659 M05661 M05662 M05669 M05671 M05672 M05679 M0569 M0570 M05711 M05712 M05719 M05721 M05722 M05729 M05731 M05732 M05739 M05741 M05742 M05749 M05751 M05752 M05759 M05761 M05762 M05769 M05771 M05772 M05779 M0579 M057A M0580 M05811 M05812 M05819 M05821 M05822 M05829 M05831 M05832 M05839 M05841 M05842 M05849 M05851 M05852 M05859 M05861 M05862 M05869 M05871 M05872 M05879 M0589 M058A M059 M0600 M06011 M06012 M06019 M06021 M06022 M06029 M06031 M06032 M06039 M06041 M06042 M06049 M06051 M06052 M06059 M06061 M06062 M06069 M06071 M06072 M06079 M0608 M0609 M060A M061 M0620 M06211 M06212 M06219 M06221 M06222 M06229 M06231 M06232 M06239 M06241 M06242 M06249 M06251 M06252 M06259 M06261 M06262 M06269 M06271 M06272 M06279 M0628 M0629 M0630 M06311 M06312 M06319 M06321 M06322 M06329 M06331 M06332 M06339 M06341 M06342 M06349 M06351 M06352 M06359 M06361 M06362 M06369 M06371 M06372 M06379 M0638 M0639 M064 M0680 M06811 M06812 M06819 M06821 M06822 M06829 M06831 M06832 M06839 M06841 M06842 M06849 M06851 M06852 M06859 M06861 M06862 M06869 M06871 M06872 M06879 M0688 M0689 M068A M069 M0760 M07611 M07612 M07619 M07621 M07622 M07629 M07631 M07632 M07639 M07641 M07642 M07649 M07651 M07652 M07659 M07661 M07662 M07669 M07671 M07672 M07679 M0768 M0769 M0800 M08011 M08012 M08019 M08021 M08022 M08029 M08031 M08032 M08039 M08041 M08042 M08049 M08051 M08052 M08059 M08061 M08062 M08069 M08071 M08072 M08079 M0808 M0809 M080A M081 M0820 M08211 M08212 M08219 M08221 M08222 M08229 M08231 M08232 M08239 M08241 M08242 M08249 M08251 M08252 M08259 M08261 M08262 M08269 M08271 M08272 M08279 M0828 M0829 M082A M083 M0840 M08411 M08412 M08419 M08421 M08422 M08429 M08431 M08432 M08439 M08441 M08442 M08449 M08451 M08452 M08459 M08461 M08462 M08469 M08471 M08472 M08479 M0848 M084A M0880 M08811 M08812 M08819 M08821 M08822 M08829 M08831 M08832 M08839 M08841 M08842 M08849 M08851 M08852 M08859 M08861 M08862 M08869 M08871 M08872 M08879 M0888 M0889 M0890 M08911 M08912 M08919 M08921 M08922 M08929 M08931 M08932 M08939 M08941 M08942 M08949 M08951 M08952 M08959 M08961 M08962 M08969 M08971 M08972 M08979 M0898 M0899 M089A M1200 M12011 M12012 M12019 M12021 M12022 M12029 M12031 M12032 M12039 M12041 M12042 M12049 M12051 M12052 M12059 M12061 M12062 M12069 M12071 M12072 M12079 M1208 M1209 M300 M301 M302 M303 M308 M310 M311 M3110 M3111 M3119 M312 M3130 M3131 M314 M315 M316 M317 M318 M319 M320 M3210 M3211 M3212 M3213 M3214 M3215 M3219 M328 M329 M3300 M3301 M3302 M3303 M3309 M3310 M3311 M3312 M3313 M3319 M3320 M3321 M3322 M3329 M3390 M3391 M3392 M3393 M3399 M340 M341 M342 M3481 M3482 M3483 M3489 M349 M3500 M3501 M3502 M3503 M3504 M3505 M3506 M3507 M3508 M3509 M350A M350B M350C M351 M352 M353 M355 M356 M358 M3581 M3589 M359 M360 M361 M368 M450 M451 M452 M453 M454 M455 M456 M457 M458 M459 M45A0 M45A1 M45A2 M45A3 M45A4 M45A5 M45A6 M45A7 M45A8 M45AB |
| Chronic blood loss anemia | D500 O9081 O9902 O9903 |
| Leukemia | C9010 C9011 C9012 C9100 C9101 C9102 C9110 C9111 C9112 C9130 C9131 C9132 C9140 C9141 C9142 C9150 C9151 C9152 C9160 C9161 C9162 C9190 C9191 C9192 C91A0 C91A1 C91A2 C91Z0 C91Z1 C91Z2 C9200 C9201 C9202 C9210 C9211 C9212 C9220 C9221 C9222 C9230 C9231 C9232 C9240 C9241 C9242 C9250 C9251 C9252 C9260 C9261 C9262 C9290 C9291 C9292 C92A0 C92A1 C92A2 C92Z0 C92Z1 C92Z2 C9300 C9301 C9302 C9310 C9311 C9312 C9330 C9331 C9332 C9390 C9391 C9392 C93Z0 C93Z1 C93Z2 C9400 C9401 C9402 C9420 C9421 C9422 C9430 C9431 C9432 C9440 C9441 C9442 C946 C9480 C9481 C9482 C9500 C9501 C9502 C9510 C9511 C9512 C9590 C9591 C9592 |
| Lymphoma | C8100 C8101 C8102 C8103 C8104 C8105 C8106 C8107 C8108 C8109 C8110 C8111 C8112 C8113 C8114 C8115 C8116 C8117 C8118 C8119 C8120 C8121 C8122 C8123 C8124 C8125 C8126 C8127 C8128 C8129 C8130 C8131 C8132 C8133 C8134 C8135 C8136 C8137 C8138 C8139 C8140 C8141 C8142 C8143 C8144 C8145 C8146 C8147 C8148 C8149 C8170 C8171 C8172 C8173 C8174 C8175 C8176 C8177 C8178 C8179 C8190 C8191 C8192 C8193 C8194 C8195 C8196 C8197 C8198 C8199 C8200 C8201 C8202 C8203 C8204 C8205 C8206 C8207 C8208 C8209 C8210 C8211 C8212 C8213 C8214 C8215 C8216 C8217 C8218 C8219 C8220 C8221 C8222 C8223 C8224 C8225 C8226 C8227 C8228 C8229 C8230 C8231 C8232 C8233 C8234 C8235 C8236 C8237 C8238 C8239 C8240 C8241 C8242 C8243 C8244 C8245 C8246 C8247 C8248 C8249 C8250 C8251 C8252 C8253 C8254 C8255 C8256 C8257 C8258 C8259 C8260 C8261 C8262 C8263 C8264 C8265 C8266 C8267 C8268 C8269 C8280 C8281 C8282 C8283 C8284 C8285 C8286 C8287 C8288 C8289 C8290 C8291 C8292 C8293 C8294 C8295 C8296 C8297 C8298 C8299 C8300 C8301 C8302 C8303 C8304 C8305 C8306 C8307 C8308 C8309 C8310 C8311 C8312 C8313 C8314 C8315 C8316 C8317 C8318 C8319 C8330 C8331 C8332 C8333 C8334 C8335 C8336 C8337 C8338 C8339 C8350 C8351 C8352 C8353 C8354 C8355 C8356 C8357 C8358 C8359 C8370 C8371 C8372 C8373 C8374 C8375 C8376 C8377 C8378 C8379 C8380 C8381 C8382 C8383 C8384 C8385 C8386 C8387 C8388 C8389 C8390 C8391 C8392 C8393 C8394 C8395 C8396 C8397 C8398 C8399 C8400 C8401 C8402 C8403 C8404 C8405 C8406 C8407 C8408 C8409 C8410 C8411 C8412 C8413 C8414 C8415 C8416 C8417 C8418 C8419 C8440 C8441 C8442 C8443 C8444 C8445 C8446 C8447 C8448 C8449 C8460 C8461 C8462 C8463 C8464 C8465 C8466 C8467 C8468 C8469 C8470 C8471 C8472 C8473 C8474 C8475 C8476 C8477 C8478 C8479 C847A C8490 C8491 C8492 C8493 C8494 C8495 C8496 C8497 C8498 C8499 C84A0 C84A1 C84A2 C84A3 C84A4 C84A5 C84A6 C84A7 C84A8 C84A9 C84Z0 C84Z1 C84Z2 C84Z3 C84Z4 C84Z5 C84Z6 C84Z7 C84Z8 C84Z9 C8510 C8511 C8512 C8513 C8514 C8515 C8516 C8517 C8518 C8519 C8520 C8521 C8522 C8523 C8524 C8525 C8526 C8527 C8528 C8529 C8580 C8581 C8582 C8583 C8584 C8585 C8586 C8587 C8588 C8589 C8590 C8591 C8592 C8593 C8594 C8595 C8596 C8597 C8598 C8599 C860 C861 C862 C863 C864 C865 C866 C880 C882 C883 C884 C888 C889 C9000 C9001 C9002 C9020 C9021 C9022 C9030 C9031 C9032 C960 C962 C9620 C9621 C9622 C9629 C964 C969 C96A C96Z D47Z9 |
| Metastatic Cancer | C770 C771 C772 C773 C774 C775 C778 C779 C7800 C7801 C7802 C781 C782 C7830 C7839 C784 C785 C786 C787 C7880 C7889 C7900 C7901 C7902 C7910 C7911 C7919 C792 C7931 C7932 C7940 C7949 C7951 C7952 C7960 C7961 C7962 C7963 C7970 C7971 C7972 C7981 C7982 C7989 C799 C7B00 C7B01 C7B02 C7B03 C7B04 C7B09 C7B1 C7B8 C800 |
| Solid tumor without metastases, in situ | D0000 D0001 D0002 D0003 D0004 D0005 D0006 D0007 D0008 D001 D002 D010 D011 D012 D013 D0140 D0149 D015 D017 D019 D020 D021 D0220 D0221 D0222 D023 D024 D030 D0310 D0311 D03111 D03112 D0312 D03121 D03122 D0320 D0321 D0322 D0330 D0339 D034 D0351 D0352 D0359 D0360 D0361 D0362 D0370 D0371 D0372 D038 D039 D040 D0410 D0411 D04111 D04112 D0412 D04121 D04122 D0420 D0421 D0422 D0430 D0439 D044 D045 D0460 D0461 D0462 D0470 D0471 D0472 D048 D049 D0500 D0501 D0502 D0510 D0511 D0512 D0580 D0581 D0582 D0590 D0591 D0592 D060 D061 D067 D069 D070 D071 D072 D0730 D0739 D074 D075 D0760 D0761 D0769 D090 D0910 D0919 D0920 D0921 D0922 D093 D098 D099 |
| Solid tumor without metastasis, malignant | C000 C001 C002 C003 C004 C005 C006 C008 C009 C01 C020 C021 C022 C023 C024 C028 C029 C030 C031 C039 C040 C041 C048 C049 C050 C051 C052 C058 C059 C060 C061 C062 C0680 C0689 C069 C07 C080 C081 C089 C090 C091 C098 C099 C100 C101 C102 C103 C104 C108 C109 C110 C111 C112 C113 C118 C119 C12 C130 C131 C132 C138 C139 C140 C142 C148 C153 C154 C155 C158 C159 C160 C161 C162 C163 C164 C165 C166 C168 C169 C170 C171 C172 C173 C178 C179 C180 C181 C182 C183 C184 C185 C186 C187 C188 C189 C19 C20 C210 C211 C212 C218 C220 C221 C222 C223 C224 C227 C228 C229 C23 C240 C241 C248 C249 C250 C251 C252 C253 C254 C257 C258 C259 C260 C261 C269 C300 C301 C310 C311 C312 C313 C318 C319 C320 C321 C322 C323 C328 C329 C33 C3400 C3401 C3402 C3410 C3411 C3412 C342 C3430 C3431 C3432 C3480 C3481 C3482 C3490 C3491 C3492 C37 C380 C381 C382 C383 C384 C388 C390 C399 C4000 C4001 C4002 C4010 C4011 C4012 C4020 C4021 C4022 C4030 C4031 C4032 C4080 C4081 C4082 C4090 C4091 C4092 C410 C411 C412 C413 C414 C419 C430 C4310 C4311 C43111 C43112 C4312 C43121 C43122 C4320 C4321 C4322 C4330 C4331 C4339 C434 C4351 C4352 C4359 C4360 C4361 C4362 C4370 C4371 C4372 C438 C439 C4400 C4409 C44101 C44102 C441021 C441022 C44109 C441091 C441092 C44131 C441321 C441322 C441391 C441392 C44191 C44192 C441921 C441922 C44199 C441991 C441992 C44201 C44202 C44209 C44291 C44292 C44299 C44300 C44301 C44309 C44390 C44391 C44399 C4440 C4449 C44500 C44501 C44509 C44590 C44591 C44599 C44601 C44602 C44609 C44691 C44692 C44699 C44701 C44702 C44709 C44791 C44792 C44799 C4480 C4489 C4490 C4499 C450 C451 C452 C457 C459 C460 C461 C462 C463 C464 C4650 C4651 C4652 C467 C469 C470 C4710 C4711 C4712 C4720 C4721 C4722 C473 C474 C475 C476 C478 C479 C480 C481 C482 C488 C490 C4910 C4911 C4912 C4920 C4921 C4922 C493 C494 C495 C496 C498 C499 C49A0 C49A1 C49A2 C49A3 C49A4 C49A5 C49A9 C4A0 C4A10 C4A11 C4A111 C4A112 C4A12 C4A121 C4A122 C4A20 C4A21 C4A22 C4A30 C4A31 C4A39 C4A4 C4A51 C4A52 C4A59 C4A60 C4A61 C4A62 C4A70 C4A71 C4A72 C4A8 C4A9 C50011 C50012 C50019 C50021 C50022 C50029 C50111 C50112 C50119 C50121 C50122 C50129 C50211 C50212 C50219 C50221 C50222 C50229 C50311 C50312 C50319 C50321 C50322 C50329 C50411 C50412 C50419 C50421 C50422 C50429 C50511 C50512 C50519 C50521 C50522 C50529 C50611 C50612 C50619 C50621 C50622 C50629 C50811 C50812 C50819 C50821 C50822 C50829 C50911 C50912 C50919 C50921 C50922 C50929 C510 C511 C512 C518 C519 C52 C530 C531 C538 C539 C540 C541 C542 C543 C548 C549 C55 C561 C562 C563 C569 C5700 C5701 C5702 C5710 C5711 C5712 C5720 C5721 C5722 C573 C574 C577 C578 C579 C58 C600 C601 C602 C608 C609 C61 C6200 C6201 C6202 C6210 C6211 C6212 C6290 C6291 C6292 C6300 C6301 C6302 C6310 C6311 C6312 C632 C637 C638 C639 C641 C642 C649 C651 C652 C659 C661 C662 C669 C670 C671 C672 C673 C674 C675 C676 C677 C678 C679 C680 C681 C688 C689 C6900 C6901 C6902 C6910 C6911 C6912 C6920 C6921 C6922 C6930 C6931 C6932 C6940 C6941 C6942 C6950 C6951 C6952 C6960 C6961 C6962 C6980 C6981 C6982 C6990 C6991 C6992 C700 C701 C709 C710 C711 C712 C713 C714 C715 C716 C717 C718 C719 C720 C721 C7220 C7221 C7222 C7230 C7231 C7232 C7240 C7241 C7242 C7250 C7259 C729 C73 C7400 C7401 C7402 C7410 C7411 C7412 C7490 C7491 C7492 C750 C751 C752 C753 C754 C755 C758 C759 C760 C761 C762 C763 C7640 C7641 C7642 C7650 C7651 C7652 C768 C7A00 C7A010 C7A011 C7A012 C7A019 C7A020 C7A021 C7A022 C7A023 C7A024 C7A025 C7A026 C7A029 C7A090 C7A091 C7A092 C7A093 C7A094 C7A095 C7A096 C7A098 C7A1 C7A8 D469 E3121 E3122 E3123 |
| Cerebrovascular disease, present on admission | G450 G451 G452 G453 G454 G458 G459 G460 G461 G462 G463 G464 G465 G466 G467 G468 H3400 H3401 H3402 H3403 H3410 H3411 H3412 H3413 H34211 H34212 H34213 H34219 H34231 H34232 H34233 H34239 I6000 I6001 I6002 I6010 I6011 I6012 I602 I6020 I6021 I6022 I6030 I6031 I6032 I604 I6050 I6051 I6052 I606 I607 I608 I609 I610 I611 I612 I613 I614 I615 I616 I618 I619 I6200 I6203 I621 I629 I6300 I63011 I63012 I63013 I63019 I6302 I63031 I63032 I63033 I63039 I6309 I6310 I63111 I63112 I63113 I63119 I6312 I63131 I63132 I63133 I63139 I6319 I6320 I63211 I63212 I63213 I63219 I6322 I63231 I63232 I63233 I63239 I6329 I6330 I63311 I63312 I63313 I63319 I63321 I63322 I63323 I63329 I63331 I63332 I63333 I63339 I63341 I63342 I63343 I63349 I6339 I6340 I63411 I63412 I63413 I63419 I63421 I63422 I63423 I63429 I63431 I63432 I63433 I63439 I63441 I63442 I63443 I63449 I6349 I6350 I63511 I63512 I63513 I63519 I63521 I63522 I63523 I63529 I63531 I63532 I63533 I63539 I63541 I63542 I63543 I63549 I6359 I636 I638 I6381 I6389 I639 I6501 I6502 I6503 I6509 I651 I6521 I6522 I6523 I6529 I658 I659 I6601 I6602 I6603 I6609 I6611 I6612 I6613 I6619 I6621 I6622 I6623 I6629 I663 I668 I669 |
| Cerebrovascular disease,sequela | I6930 I6931 I69310 I69311 I69312 I69313 I69314 I69315 I69318 I69319 I69320 I69321 I69322 I69323 I69328 I69331 I69332 I69333 I69334 I69339 I69341 I69342 I69343 I69344 I69349 I69351 I69352 I69353 I69354 I69359 I69361 I69362 I69363 I69364 I69365 I69369 I69390 I69391 I69392 I69393 I69398 I6980 I6981 I69810 I69811 I69812 I69813 I69814 I69815 I69818 I69819 I69820 I69821 I69822 I69823 I69828 I69831 I69832 I69833 I69834 I69839 I69841 I69842 I69843 I69844 I69849 I69851 I69852 I69853 I69854 I69859 I69861 I69862 I69863 I69864 I69865 I69869 I69890 I69891 I69892 I69893 I69898 I6990 I6991 I69910 I69911 I69912 I69913 I69914 I69915 I69918 I69919 I69920 I69921 I69922 I69923 I69928 I69931 I69932 I69933 I69934 I69939 I69941 I69942 I69943 I69944 I69949 I69951 I69952 I69953 I69954 I69959 I69961 I69962 I69963 I69964 I69965 I69969 I69990 I69991 I69992 I69993 I69998 P91821 P91822 P91823 P91829 |
| Coagulopathy | D6109 D611 D612 D613 D61810 D61811 D61818 D6182 D6189 D619 D65 D66 D67 D680 D681 D682 D68311 D68312 D68318 D6832 D684 D688 D689 D691 D693 D6941 D6942 D6949 D6951 D6959 D696 D698 D699 D7582 O99111 O99112 O99113 O99119 O9912 O9913 |
| Dementia | F0150 F0151 F0280 F0281 F0390 F0391 G300 G301 G308 G309 G3101 G3109 G311 G312 G3181 G3182 G3183 G3185 G3189 G319 |
| Depression | F0631 F0632 F0634 F320 F321 F322 F323 F328 F3281 F3289 F329 F32A F330 F331 F332 F333 F338 F339 F341 |
| Diabetes with chronic complications | E0821 E0822 E0829 E08311 E08319 E08321 E083211 E083212 E083213 E083219 E08329 E083291 E083292 E083293 E083299 E08331 E083311 E083312 E083313 E083319 E08339 E083391 E083392 E083393 E083399 E08341 E083411 E083412 E083413 E083419 E08349 E083491 E083492 E083493 E083499 E08351 E083511 E083512 E083513 E083519 E083521 E083522 E083523 E083529 E083531 E083532 E083533 E083539 E083541 E083542 E083543 E083549 E083551 E083552 E083553 E083559 E08359 E083591 E083592 E083593 E083599 E0836 E0837X1 E0837X2 E0837X3 E0837X9 E0839 E0840 E0841 E0842 E0843 E0844 E0849 E0851 E0852 E0859 E08610 E08618 E08620 E08621 E08622 E08628 E08630 E08638 E08641 E08649 E0865 E0869 E088 E0921 E0922 E0929 E09311 E09319 E09321 E093211 E093212 E093213 E093219 E09329 E093291 E093292 E093293 E093299 E09331 E093311 E093312 E093313 E093319 E09339 E093391 E093392 E093393 E093399 E09341 E093411 E093412 E093413 E093419 E09349 E093491 E093492 E093493 E093499 E09351 E093511 E093512 E093513 E093519 E093521 E093522 E093523 E093529 E093531 E093532 E093533 E093539 E093541 E093542 E093543 E093549 E093551 E093552 E093553 E093559 E09359 E093591 E093592 E093593 E093599 E0936 E0937X1 E0937X2 E0937X3 E0937X9 E0939 E0940 E0941 E0942 E0943 E0944 E0949 E0951 E0952 E0959 E09610 E09618 E09620 E09621 E09622 E09628 E09630 E09638 E09641 E09649 E0965 E0969 E098 E1021 E1022 E1029 E10311 E10319 E10321 E103211 E103212 E103213 E103219 E10329 E103291 E103292 E103293 E103299 E10331 E103311 E103312 E103313 E103319 E10339 E103391 E103392 E103393 E103399 E10341 E103411 E103412 E103413 E103419 E10349 E103491 E103492 E103493 E103499 E10351 E103511 E103512 E103513 E103519 E103521 E103522 E103523 E103529 E103531 E103532 E103533 E103539 E103541 E103542 E103543 E103549 E103551 E103552 E103553 E103559 E10359 E103591 E103592 E103593 E103599 E1036 E1037X1 E1037X2 E1037X3 E1037X9 E1039 E1040 E1041 E1042 E1043 E1044 E1049 E1051 E1052 E1059 E10610 E10618 E10620 E10621 E10622 E10628 E10630 E10638 E10641 E10649 E1065 E1069 E108 E1121 E1122 E1129 E11311 E11319 E11321 E113211 E113212 E113213 E113219 E11329 E113291 E113292 E113293 E113299 E11331 E113311 E113312 E113313 E113319 E11339 E113391 E113392 E113393 E113399 E11341 E113411 E113412 E113413 E113419 E11349 E113491 E113492 E113493 E113499 E11351 E113511 E113512 E113513 E113519 E113521 E113522 E113523 E113529 E113531 E113532 E113533 E113539 E113541 E113542 E113543 E113549 E113551 E113552 E113553 E113559 E11359 E113591 E113592 E113593 E113599 E1136 E1137X1 E1137X2 E1137X3 E1137X9 E1139 E1140 E1141 E1142 E1143 E1144 E1149 E1151 E1152 E1159 E11610 E11618 E11620 E11621 E11622 E11628 E11630 E11638 E11641 E11649 E1165 E1169 E118 E1321 E1322 E1329 E13311 E13319 E13321 E133211 E133212 E133213 E133219 E13329 E133291 E133292 E133293 E133299 E13331 E133311 E133312 E133313 E133319 E13339 E133391 E133392 E133393 E133399 E13341 E133411 E133412 E133413 E133419 E13349 E133491 E133492 E133493 E133499 E13351 E133511 E133512 E133513 E133519 E133521 E133522 E133523 E133529 E133531 E133532 E133533 E133539 E133541 E133542 E133543 E133549 E133551 E133552 E133553 E133559 E13359 E133591 E133592 E133593 E133599 E1336 E1337X1 E1337X2 E1337X3 E1337X9 E1339 E1340 E1341 E1342 E1343 E1344 E1349 E1351 E1352 E1359 E13610 E13618 E13620 E13621 E13622 E13628 E13630 E13638 E13641 E13649 E1365 E1369 E138 |
| Diabetes without chronic complications | E0800 E0801 E0810 E0811 E089 E0900 E0901 E0910 E0911 E099 E1010 E1011 E109 E1100 E1101 E1110 E1111 E119 E1300 E1301 E1310 E1311 E139 O24011 O24012 O24013 O24019 O2402 O2403 O24111 O24112 O24113 O24119 O2412 O2413 O24311 O24312 O24313 O24319 O2432 O2433 O24410 O24414 O24415 O24419 O24420 O24424 O24425 O24429 O24430 O24434 O24435 O24439 O24811 O24812 O24813 O24819 O2482 O2483 O24911 O24912 O24913 O24919 O2492 O2493 |
| Drug abuse | F1110 F1111 F11120 F11121 F11122 F11129 F1113 F1114 F11150 F11151 F11159 F11181 F11182 F11188 F1119 F1120 F1121 F11220 F11221 F11222 F11229 F1123 F1124 F11250 F11251 F11259 F11281 F11282 F11288 F1129 F1210 F1211 F12120 F12121 F12122 F12129 F1213 F12150 F12151 F12159 F12180 F12188 F1219 F1220 F1221 F12220 F12221 F12222 F12229 F1223 F12250 F12251 F12259 F12280 F12288 F1229 F1310 F1311 F13120 F13121 F13129 F13130 F13131 F13132 F13139 F1314 F13150 F13151 F13159 F13180 F13181 F13182 F13188 F1319 F1320 F1321 F13220 F13221 F13229 F13230 F13231 F13232 F13239 F1324 F13250 F13251 F13259 F1326 F1327 F13280 F13281 F13282 F13288 F1329 F1410 F1411 F14120 F14121 F14122 F14129 F1413 F1414 F14150 F14151 F14159 F14180 F14181 F14182 F14188 F1419 F1420 F1421 F14220 F14221 F14222 F14229 F1423 F1424 F14250 F14251 F14259 F14280 F14281 F14282 F14288 F1429 F1510 F1511 F15120 F15121 F15122 F15129 F1513 F1514 F15150 F15151 F15159 F15180 F15181 F15182 F15188 F1519 F1520 F1521 F15220 F15221 F15222 F15229 F1523 F1524 F15250 F15251 F15259 F15280 F15281 F15282 F15288 F1529 F1610 F1611 F16120 F16121 F16122 F16129 F1614 F16150 F16151 F16159 F16180 F16183 F16188 F1619 F1620 F1621 F16220 F16221 F16229 F1624 F16250 F16251 F16259 F16280 F16283 F16288 F1629 F1810 F1811 F18120 F18121 F18129 F1814 F18150 F18151 F18159 F1817 F18180 F18188 F1819 F1820 F1821 F18220 F18221 F18229 F1824 F18250 F18251 F18259 F1827 F18280 F18288 F1829 F1910 F1911 F19120 F19121 F19122 F19129 F19130 F19131 F19132 F19139 F1914 F19150 F19151 F19159 F1916 F1917 F19180 F19181 F19182 F19188 F1919 F1920 F1921 F19220 F19221 F19222 F19229 F19230 F19231 F19232 F19239 F1924 F19250 F19251 F19259 F1926 F1927 F19280 F19281 F19282 F19288 F1929 O99320 O99321 O99322 O99323 O99324 O99325 |
| Heart failure | I0981 I110 I130 I132 I501 I5020 I5021 I5022 I5023 I5030 I5031 I5032 I5033 I5040 I5041 I5042 I5043 I50810 I50811 I50812 I50813 I50814 I5082 I5083 I5084 I5089 I509 I5181 I97130 I97131 O29121 O29122 O29123 O29129 R570 Z95811 Z95812 |
| Hypertension, complicated | H35031 H35032 H35033 H35039 I110 I119 I120 I129 I130 I1310 I1311 I132 I150 I151 I152 I158 I159 I161 I674 O10111 O10112 O10113 O10119 O1012 O1013 O10211 O10212 O10213 O10219 O1022 O1023 O10311 O10312 O10313 O10319 O1032 O1033 O10411 O10412 O10413 O10419 O1042 O1043 O10911 O10912 O10913 O10919 O1092 O1093 O111 O112 O113 O114 O115 O119 O161 O162 O163 O164 O165 O169 |
| Hypertension, uncomplicated | I10 I160 I169 O10011 O10012 O10013 O10019 O1002 O1003 |
| Liver disease, mild | A5145 A5274 B180 B181 B182 B188 B189 B1910 B1920 B199 B251 B581 K700 K7010 K7011 K702 K7030 K7031 K709 K713 K714 K7150 K7151 K716 K717 K718 K730 K731 K732 K738 K739 K740 K7400 K7401 K7402 K741 K742 K743 K744 K745 K7460 K7469 K751 K752 K753 K754 K7581 K7589 K759 K760 K761 K762 K763 K764 K7681 K7689 K769 K77 |
| Liver disease, severe | B190 B1911 B1921 I8500 I8501 I8510 I8511 I864 K7040 K7041 K7210 K7211 K7290 K7291 K765 K766 K767 K9182 Z944 |
| Chronic pulmonary disease | J410 J411 J418 J42 J430 J431 J432 J438 J439 J440 J441 J449 J4520 J4521 J4522 J4530 J4531 J4532 J4540 J4541 J4542 J4550 J4551 J4552 J45901 J45902 J45909 J45990 J45991 J45998 J470 J471 J479 J60 J61 J620 J628 J630 J631 J632 J633 J634 J635 J636 J64 J65 J660 J661 J662 J668 J670 J671 J672 J673 J674 J675 J676 J677 J678 J679 J684 J701 J703 |
| Neurological disorders affecting movement | G08 G10 G110 G111 G1110 G1111 G1119 G112 G113 G114 G118 G119 G120 G121 G1220 G1221 G1222 G1223 G1224 G1225 G1229 G128 G129 G130 G131 G132 G138 G20 G210 G2111 G2119 G212 G213 G214 G218 G219 G230 G231 G232 G238 G239 G2409 G241 G242 G248 G254 G255 G2570 G2571 G2579 G2581 G2582 G2583 G2589 G259 G26 G320 G3281 G3289 G803 |
| Other neurological disorders | E7500 E7501 E7502 E7509 E7510 E7511 E7519 E7523 E7525 E7526 E7529 E754 F05 F842 G35 G360 G368 G369 G370 G371 G372 G373 G374 G375 G378 G379 G47411 G47419 G47421 G47429 G890 G910 G911 G912 G913 G914 G918 G919 G930 G9340 G9341 G9349 G935 G936 G937 G9381 G9382 G9389 G939 G94 O99350 O99351 O99352 O99353 O99354 O99355 P9160 P9161 P9162 P9163 |
| Seizures and epilepsy | G40001 G40009 G40011 G40019 G40101 G40109 G40111 G40119 G40201 G40209 G40211 G40219 G40301 G40309 G40311 G40319 G40401 G40409 G40411 G40419 G4042 G40501 G40509 G40801 G40802 G40803 G40804 G40811 G40812 G40813 G40814 G40821 G40822 G40823 G40824 G40833 G40834 G4089 G40901 G40909 G40911 G40919 G40A01 G40A09 G40A11 G40A19 G40B01 G40B09 G40B11 G40B19 R561 R569 |
| Obesity | E6601 E6609 E661 E662 E668 E669 O99210 O99211 O99212 O99213 O99214 O99215 R939 Z6830 Z6831 Z6832 Z6833 Z6834 Z6835 Z6836 Z6837 Z6838 Z6839 Z6841 Z6842 Z6843 Z6844 Z6845 Z6854 |
| Paralysis | G041 G800 G801 G802 G808 G809 G8100 G8101 G8102 G8103 G8104 G8110 G8111 G8112 G8113 G8114 G8190 G8191 G8192 G8193 G8194 G8220 G8221 G8222 G8250 G8251 G8252 G8253 G8254 G830 G8310 G8311 G8312 G8313 G8314 G8320 G8321 G8322 G8323 G8324 G8330 G8331 G8332 G8333 G8334 G834 G835 G8381 G8382 G8383 G8384 G8389 G839 I69031 I69032 I69033 I69034 I69039 I69041 I69042 I69043 I69044 I69049 I69051 I69052 I69053 I69054 I69059 I69061 I69062 I69063 I69064 I69065 I69069 I69131 I69132 I69133 I69134 I69139 I69141 I69142 I69143 I69144 I69149 I69151 I69152 I69153 I69154 I69159 I69161 I69162 I69163 I69164 I69165 I69169 I69231 I69232 I69233 I69234 I69239 I69241 I69242 I69243 I69244 I69249 I69251 I69252 I69253 I69254 I69259 I69261 I69262 I69263 I69264 I69265 I69269 I69331 I69332 I69333 I69334 I69339 I69341 I69342 I69343 I69344 I69349 I69351 I69352 I69353 I69354 I69359 I69361 I69362 I69363 I69364 I69365 I69369 I69831 I69832 I69833 I69834 I69839 I69841 I69842 I69843 I69844 I69849 I69851 I69852 I69853 I69854 I69859 I69861 I69862 I69863 I69864 I69865 I69869 I69931 I69932 I69933 I69934 I69939 I69941 I69942 I69943 I69944 I69949 I69951 I69952 I69953 I69954 I69959 I69961 I69962 I69963 I69964 I69965 I69969 R532 |
| Peripheral vascular disease | A5200 A5201 A5202 A5209 I700 I701 I70201 I70202 I70203 I70208 I70209 I70211 I70212 I70213 I70218 I70219 I70221 I70222 I70223 I70228 I70229 I70231 I70232 I70233 I70234 I70235 I70238 I70239 I70241 I70242 I70243 I70244 I70245 I70248 I70249 I7025 I70261 I70262 I70263 I70268 I70269 I70291 I70292 I70293 I70298 I70299 I70301 I70302 I70303 I70308 I70309 I70311 I70312 I70313 I70318 I70319 I70321 I70322 I70323 I70328 I70329 I70331 I70332 I70333 I70334 I70335 I70338 I70339 I70341 I70342 I70343 I70344 I70345 I70348 I70349 I7035 I70361 I70362 I70363 I70368 I70369 I70391 I70392 I70393 I70398 I70399 I70401 I70402 I70403 I70408 I70409 I70411 I70412 I70413 I70418 I70419 I70421 I70422 I70423 I70428 I70429 I70431 I70432 I70433 I70434 I70435 I70438 I70439 I70441 I70442 I70443 I70444 I70445 I70448 I70449 I7045 I70461 I70462 I70463 I70468 I70469 I70491 I70492 I70493 I70498 I70499 I70501 I70502 I70503 I70508 I70509 I70511 I70512 I70513 I70518 I70519 I70521 I70522 I70523 I70528 I70529 I70531 I70532 I70533 I70534 I70535 I70538 I70539 I70541 I70542 I70543 I70544 I70545 I70548 I70549 I7055 I70561 I70562 I70563 I70568 I70569 I70591 I70592 I70593 I70598 I70599 I70601 I70602 I70603 I70608 I70609 I70611 I70612 I70613 I70618 I70619 I70621 I70622 I70623 I70628 I70629 I70631 I70632 I70633 I70634 I70635 I70638 I70639 I70641 I70642 I70643 I70644 I70645 I70648 I70649 I7065 I70661 I70662 I70663 I70668 I70669 I70691 I70692 I70693 I70698 I70699 I70701 I70702 I70703 I70708 I70709 I70711 I70712 I70713 I70718 I70719 I70721 I70722 I70723 I70728 I70729 I70731 I70732 I70733 I70734 I70735 I70738 I70739 I70741 I70742 I70743 I70744 I70745 I70748 I70749 I7075 I70761 I70762 I70763 I70768 I70769 I70791 I70792 I70793 I70798 I70799 I708 I7090 I7091 I7092 I7100 I7101 I7102 I7103 I711 I712 I713 I714 I715 I716 I718 I719 I720 I721 I722 I723 I724 I725 I726 I728 I729 I7301 I731 I7381 I7389 I739 I7401 I7409 I7410 I7411 I7419 I742 I743 I744 I745 I748 I749 I75011 I75012 I75013 I75019 I75021 I75022 I75023 I75029 I7581 I7589 I770 I771 I772 I773 I774 I775 I776 I7770 I7771 I7772 I7773 I7774 I7775 I7776 I7777 I7779 I77810 I77811 I77812 I77819 I7789 I779 I780 I781 I788 I789 I790 I791 I798 K551 Z95820 Z95828 |
| Psychoses | F060 F061 F062 F0630 F0633 F11150 F11151 F11159 F11250 F11251 F11259 F11950 F11951 F11959 F12150 F12151 F12159 F12250 F12251 F12259 F12950 F12951 F12959 F13150 F13151 F13159 F13250 F13251 F13259 F13950 F13951 F13959 F14150 F14151 F14159 F14250 F14251 F14259 F14950 F14951 F14959 F15150 F15151 F15159 F15250 F15251 F15259 F15950 F15951 F15959 F16150 F16151 F16159 F16250 F16251 F16259 F16950 F16951 F16959 F18150 F18151 F18159 F18250 F18251 F18259 F18950 F18951 F18959 F19150 F19151 F19159 F19250 F19251 F19259 F19950 F19951 F19959 F200 F201 F202 F203 F205 F2081 F2089 F209 F21 F22 F23 F24 F250 F251 F258 F259 F28 F29 F3010 F3011 F3012 F3013 F302 F303 F304 F308 F309 F310 F3110 F3111 F3112 F3113 F312 F3130 F3131 F3132 F314 F315 F3160 F3161 F3162 F3163 F3164 F3170 F3171 F3172 F3173 F3174 F3175 F3176 F3177 F3178 F3181 F3189 F319 F324 F325 F3340 F3341 F3342 F340 F348 F3481 F3489 F349 F39 F4489 F843 |
| Pulmonary circulation disease | I270 I271 I272 I2720 I2721 I2722 I2723 I2724 I2729 I2781 I2782 I2783 I2789 I279 I280 I281 I288 I289 |
| Renal failure, moderate | N183 N1830 N1831 N1832 N189 N19 |
| Renal failure, severe | I120 I1311 I132 N184 N185 N186 Z4901 Z4902 Z4931 Z4932 Z9115 Z940 Z992 |
| Hypothyroidism | E000 E001 E002 E009 E010 E011 E012 E018 E02 E030 E031 E032 E033 E034 E035 E038 E039 E890 |
| Other thyroid disorders | E040 E041 E042 E048 E049 E0500 E0501 E0510 E0511 E0520 E0521 E0530 E0531 E0540 E0541 E0580 E0581 E0590 E0591 E060 E061 E062 E063 E064 E065 E069 O905 |
| Peptic ulcer with bleeding | K250 K251 K252 K253 K254 K255 K256 K257 K259 K260 K261 K262 K263 K264 K265 K266 K267 K269 K270 K271 K272 K273 K274 K275 K276 K277 K279 K280 K281 K282 K283 K284 K285 K286 K287 K289 |
| Valvular disease | A1884 A3282 A3951 A5203 B3321 B376 I011 I018 I019 I020 I050 I051 I052 I058 I059 I060 I061 I062 I068 I069 I070 I071 I072 I078 I079 I080 I081 I082 I083 I088 I089 I091 I0989 I330 I339 I340 I341 I342 I348 I349 I350 I351 I352 I358 I359 I360 I361 I362 I368 I369 I370 I371 I372 I378 I379 I38 I39 M3211 Q220 Q221 Q222 Q223 Q224 Q225 Q226 Q228 Q229 Q230 Q231 Q232 Q233 Q234 Q238 Q239 T8201XA T8201XD T8201XS T8202XA T8202XD T8202XS T8203XA T8203XD T8203XS T8209XA T8209XD T8209XS T82221A T82221D T82221S T82222A T82222D T82222S T82223A T82223D T82223S T82228A T82228D T82228S T826XXA T826XXD T826XXS Z952 Z953 Z954 |
| Weight loss | E40 E41 E42 E43 E440 E441 E45 E46 E640 O2510 O2511 O2512 O2513 O252 O253 R634 R64 |

**Supplementary Table 4.** Sensitivity Analysis Stratified by Mode of Delivery

Table 4a. Cesarean Delivery

| **Outcome** | **Adjusted RR (95% CI)** | | **p-value** |
| --- | --- | --- | --- |
|  | **LLP** | **PP** |  |
| **Hemorrhage** | 6.59 (6.30–6.89) | 11.43 (11.24–11.63) | <0.001 |
| **SMM any** | 1.68 (1.52–1.85) | 2.66 (2.55–2.77) | <0.001 |
| **SMM excluding transfusion** | 1.31 (1.12–1.53) | 1.66 (1.54–1.79) | <0.001 |
| **Maternal transfusion** | 2.03 (1.81–2.28) | 3.66 (3.49–3.84) | <0.001 |
| **DIC** | 1.73 (1.27–2.34) | 2.25 (1.93–2.61) | <0.001 |
| **Shock** | 1.75 (1.04–2.96) | 4.11 (3.39–4.99) | <0.001 |
| **VTE or PE** | 2.85 (1.84–4.42) | 1.98 (1.48–2.65) | <0.001 |

**Reference group: normal placentation*

Table 4b. Vaginal Delivery

| **Outcome** | **Adjusted RR (95% CI)** | | **p-value** |
| --- | --- | --- | --- |
|  | **LLP** | **PP** |  |
| **Hemorrhage** | 3.66 (3.46–3.88) | NA | <0.001 |
| **SMM any** | 2.23 (1.90–2.62) | NA | <0.001 |
| **SMM excluding transfusion** | 1.65 (1.24–2.20) | NA | 0.001 |
| **Maternal transfusion** | 2.76 (2.29–3.32) | NA | <0.001 |
| **DIC** | 2.13 (1.36–3.34) | NA | 0.001 |
| **Shock** | 2.59 (0.97–6.91) | NA | 0.057 |
| **VTE or PE** | 1.79 (0.80–3.98) | NA | 0.155 |

**Reference group: normal placentation*
